# Establishment of experimental salivary gland cancer models using organoid culture and patient-derived xenografting

**DOI:** 10.1007/s13402-022-00758-6

**Published:** 2022-12-20

**Authors:** Yoshihiro Aizawa, Kentaro Takada, Jun Aoyama, Daisuke Sano, Shoji Yamanaka, Masahide Seki, Yuta Kuze, Jordan A. Ramilowski, Ryo Okuda, Yasuharu Ueno, Yusuke Nojima, Yoshiaki Inayama, Hiromitsu Hatakeyama, Takashi Hatano, Hideaki Takahashi, Goshi Nishimura, Satoshi Fujii, Yutaka Suzuki, Hideki Taniguchi, Nobuhiko Oridate

**Affiliations:** 1 Department of Otorhinolaryngology, Head and Neck Surgery, School of Medicine, Yokohama City University, 3-9 Fukuura, Kanazawa-Ku, Yokohama, 236-0004 Japan; 2 Department of Pathology, Yokohama City University Hospital, Yokohama, Kanagawa Japan; 3 Department of Computational Biology and Medical Sciences, Graduate School of Frontier Sciences, The University of Tokyo, Kashiwa, Chiba Japan; 4 Advanced Medical Research Center, Yokohama City University, Kanagawa, Japan; 5 Roche Innovation Center, Roche Institute for Translational Bioengineering, Roche Pharma Research and Early Development, Basel, Switzerland; 6 Division of Regenerative Medicine, Center for Stem Cell Biology and Regenerative Medicine, The Institute of Medical Science, the University of Tokyo, Tokyo, Japan; 7 Department of Pathology, Yokohama City University Medical Center, Yokohama, Kanagawa Japan; 8 Department of Otolaryngology, Yokohama City University Graduate School of Medicine, Yokohama, Kanagawa Japan; 9 Department of Molecular Pathology, Yokohama City University Graduate School of Medicine, Yokohama, Kanagawa Japan

**Keywords:** Salivary gland cancer, Organoid culture, Patient derived xenograft (PDX), Patient-derived organoid (PDO), PDX-derived organoid (PDXO)

## Abstract

**Purpose:**

Depending on its histological subtype, salivary gland carcinoma (SGC) may have a poor prognosis. Due to the scarcity of preclinical experimental models, its molecular biology has so far remained largely unknown, hampering the development of new treatment modalities for patients with these malignancies. The aim of this study was to generate experimental human SGC models of multiple histological subtypes using patient-derived xenograft (PDX) and organoid culture techniques.

**Methods:**

Tumor specimens from surgically resected SGCs were processed for the preparation of PDXs and patient-derived organoids (PDOs). Specimens from SGC PDXs were also processed for PDX-derived organoid (PDXO) generation. In vivo tumorigenicity was assessed using orthotopic transplantation of SGC organoids. The pathological characteristics of each model were compared to those of the original tumors using immunohistochemistry. RNA-seq was used to analyze the genetic traits of our models.

**Results:**

Three series of PDOs, PDXs and PDXOs of salivary duct carcinomas, one series of PDOs, PDXs and PDXOs of mucoepidermoid carcinomas and PDXs of myoepithelial carcinomas were successfully generated. We found that PDXs and orthotopic transplants from PDOs/PDXOs showed similar histological features as the original tumors. Our models also retained their genetic traits, i.e., transcription profiles, genomic variants and fusion genes of the corresponding histological subtypes.

**Conclusion:**

We report the generation of SGC PDOs, PDXs and PDXOs of multiple histological subtypes, recapitulating the histological and genetical characteristics of the original tumors. These experimental SGC models may serve as a useful resource for the development of novel therapeutic strategies and for investigating the molecular mechanisms underlying the development of these malignancies.

**Supplementary Information:**

The online version contains supplementary material available at 10.1007/s13402-022-00758-6.

## Introduction

Salivary gland carcinomas (SGCs) are uncommon malignancies, representing approximately 0.3% of all cancers, with an estimated annual incidence of 0.05 to 2 per 100,000 [1]. These malignancies exhibit considerable pathologic, biological, and clinical diversity. Currently, there are 22 histological subtypes [2], which seriously hampers their accurate preoperative diagnosis [3, 4]. Among the many histological subtypes, salivary duct carcinoma (SDC) is a highly malignant one with a high rate of distant metastatic recurrence and a 5-year survival rate of only 40% [5, 6]. Adenoid cystic carcinoma (ACC) is one of the most common histological subtypes of SGC, characterized by a high rate of perineural invasion and local recurrence and a delayed onset of distant metastases [7, 8]. The prognosis of mucoepidermoid carcinoma (MEC), another common subtype of SGC, is highly dependent on its pathological grade, with a 5-year survival rate of 22.5% of high-grade cases. A majority of patients dies from distant metastasis rather than from local recurrence [9, 10]. Thus, patients diagnosed with aggressive histological subtypes of SGC have a poor prognosis.

Overall, the pathogenesis of SGCs has remained unclear [11], except for cases harboring tumor-specific recurrent chromosomal translocations that result in the formation of fusion genes, such as *CRTC1* [*MECT1*]-*MAML2* in MEC [12] or *MYB*-*NFIB* in ACC [13]. The lack of in vitro and in vivo SGC models that can effectively recapitulate the diversity of human SGCs has hampered our understanding of the mechanisms underlying their development and progression.

Recently, patient-derived xenograft (PDX) and organoid cultures have emerged as useful preclinical tools to overcome the limits of traditional two-dimensional cultures by mimicking the traits and heterogeneity of the original tumors [14, 15]. These technologies have the potential to serve as stepping stones to personalized medicine [16, 17]. Previously, we reported the establishment of ACC patient-derived organoids (PDOs), short-term organoids from ACC PDX (PDXOs of ACC) and organoids-transplanted animal models of ACC, reproducing the histological characteristics of the original tumors, and showed the significance of these models for drug screening [18]. Here, we established multiple experimental models of SGCs, including SDC, MEC and myoepithelial carcinoma (MYEC), using organoid cultures and patient-derived xenografting. Additionally, we genetically characterized these novel SGC models using RNA-seq analysis.

## Materials and methods

### Human specimens

We obtained 40 fresh SGC tumor tissues from patients undergoing surgical resection at Yokohama City University Hospital or Yokohama City University Medical Center (Yokohama, Japan) and stored them in culture medium on ice until further use (< 12 h). The pathological diagnosis for each case was confirmed by independent pathologists after sample collection. We obtained written informed consent from all patients prior to surgery.

### Patient-derived xenografts (PDXs)

PDXs were established by subcutaneous implantation of fresh minced tumors into NOD Cg‐Prkdc^scid^ Il2rg^tm1Wjl^/SzJ (NSG) mice as previously described [18]. All mice were dissected to visually examine the occurrence of metastases in lung, liver and the abdominal cavity.

### Organoid cultures

Organoid cultures from patient specimens and PDXs were performed as previously described [18, 20, 21]. Briefly, the tissue, cut into 2–4 mm pieces, was enzymatically digested with Liberase TM Research Grade (Sigma Aldrich, St. Louis, MO, USA) and Hyaluronidase (Sigma Aldrich) for 30–60 min at 37 °C. Processed tissue was passed through a 70 μm cell strainer (Corning Incorporated, Corning, NY, USA) to eliminate macroscopic pieces. The isolated cells were suspended in complete medium and seeded on growth-factor-reduced (GFR) Matrigel (Corning Incorporated) coated plates, pre-prepared as a lower layer as previously described [18]. In this procedure, 6, 12, 24 and 48 well plates were used according to cell quantity. After incubation for 16–24 h, the medium was removed and the organoids formed on the lower layer were covered with an additional layer of GFR Matrigel. Complete medium was added after the formation of a solid coating, after which the medium was changed every 2–3 days. For passaging, Matrigel containing organoids were collected from the plate and digested with TrypLE Express Enzyme (Thermo Fisher Scientific, Waltham, MA, USA) for ~ 5 to 10 min. Next, the isolated organoids were suspended in DMEM/F12 medium and physically crushed into smaller cell clumps by pipetting. These clumps were centrifuged, re-suspended in complete medium with 10 μM ROCK inhibitor (Y-27632, Sigma) and embedded in GRF Matrigel as described above. Organoids were passaged at a 1:2 to 1:1.5 dilution ratio every 2–3 weeks. To prepare frozen stocks, organoids were isolated and suspended in CELLBANKER 1 (TAKARA-BIO, Kusatsu, Shiga, Japan) and stored at − 80 °C or in liquid nitrogen. The stocks could successfully be recovered for up to at least 6 months after freezing. STR analysis was performed at BEX. CO., LTD. (Tokyo, Japan) to authenticate the identity of the organoids and their corresponding patient tissues. To check for contamination of mouse cells, we performed PCR of animal species-specific mitochondrial DNA sequences [19] using the primers listed in Supplementary Table S3.

### Transplantation of organoids

For orthotopic transplantation of SGC organoids, they were injected into the submandibular glands of NSG mice as previously described [18] at a density of 0.5 × 10^6^ to 1 × 10^6^ cells suspended in a mixture of DMEM/F12 and Matrigel. For subcutaneous transplantation of SGC organoids, organoid suspensions (1 to 2 × 10^6^ cells) were similarly injected subcutaneously into the flanks in NSG mice. Tumor volumes were measured weekly. Xenografts were harvested when the tumor diameter reached > 1 cm or 6 months after implantation and fixed for 24 h in 10% formalin.

### Immunohistochemistry

Fresh PDXs and orthotopically transplanted organoids were fixed in 10% formalin for 24 h and then embedded in paraffin following standard histological procedures. Organoids were isolated by digesting Matrigel using dispase (Sigma) for 30 min at 37 °C and embedded into a gel using iPGell (GenoStaff, Tokyo, Japan) according to the manufacturer’s protocol. Next, the organoids were fixed in 10% formalin for 24 h and paraffin-embedded. Hematoxylin–eosin staining and immunohistochemistry (IHC) were performed using standard protocols on 5 μm thick paraffin sections. The following antibodies were used for IHC: anti-human-Androgen Receptor (AR441, Dako, Carpinteria, CA, USA) 1:500, anti-pan keratin AE1/AE3/PCK26 (Roche, Basel, Switzerland) 1:1, anti-HER2 (4B5, Roche) 1:1, anti-alpha-smooth muscle actin (S131, Leica Biosystems, Buffalo Grove, IL, USA) 1:1, anti-p63 (4A4, Biocare medical, Concord, CA, USA) 1:200, anti-S-100 (Roche) 1:1000, and anti-GCDFP15 (D6, Biocare medical) 1:400. Images were acquired using an OLYMPUS BX41 microscope.

### DNA/RNA extraction

Organoids were recovered from Matrigel using TrypLe. Total RNA was extracted from organoids using TRIzol (Thermo Fisher), followed by isolation and precipitation in chloroform and 70% ethanol, and purification via column-based separation using a RNeasy Mini Kit (QIAGEN, Valencia, CA, USA). DNA was extracted from organoids using a DNA mini kit (QIAGEN) according to the manufacturer’s protocol. PDX tissues harvested from mice, as well as tissue fragments of primary salivary gland tumors, were physically homogenized using a plastic homogenizer pestle. RNA and DNA extraction from these homogenized tissues were processed similarly as described above. STR analysis was performed as described above.

### RNA-seq

RNA sequencing was performed at the Laboratory of Systems Genomics, Department of Computational Biology and Medical Sciences, at the University of Tokyo (Chiba, Japan). RNA quality and quantity were assessed using an Agilent Bioanalyzer 2100. Libraries for sequencing were constructed using TruSeq Stranded mRNA (Illumina, San Diego, CA, USA) according to the manufacturer’s protocol, followed by sequencing on an Illumina NovaSeq6000 platform to generate 70 million paired-end reads of 150 bases. The RNA-seq data are available at the DNA Data Bank of Japan Sequence Read Archive (DRA) under accession number DRA011243.

### Gene expression analysis

RNA-seq reads were quality checked and adapter trimmed using fastp (v0.20.1) [22]. Since RNA-seq reads derived from PDX tumors and PDXOs both essentially contain mouse reads, we distinguished the trimmed reads into those of humans (GRCh38/hg38) or mice (GRCm38/mm10) using xenome (v1.0.0) [23]. Only human reads were used for subsequent processing. Mouse reads and indistinguishable reads were discarded. To ensure consistency in process sampling, samples that do not contain intrinsic mouse reads, such as patient-derived organoids, were processed in the same manner as described above. The human reads were aligned to the human genome reference sequence (GRCh38/hg38) using STAR (v2.7.5c) [24] and counted for each gene using featureCounts (v2.0.1) [25]. The RNA-seq coverage and quality statistics are summarized in Supplementary Table S3. For a heatmap, hierarchical clustering analysis with complete linkage and Euclidean distance, and correlation analysis, the raw read counts per gene with at least an average of 5 counts were TMM normalized using edgeR (v3.30.3) [26] and log2-transformed. The heatmap and clustering analysis were visualized with the top 2000 variable genes using R package “pheatmap”. These 2000 genes are listed in Supplementary Table S4. The Pearson’s correlation coefficients were calculated for all genes. For a principal component analysis (PCA), we combined our samples with RNA-seq datasets of multiple salivary gland cancers downloaded from public databases. SRP067524 (including 42 samples of ACC and 5 samples of normal salivary gland), SRP067827 (including 3 samples of acinic cell carcinoma), SRP096726 (including 16 samples of SDC) and SRP109264 (including 40 samples of MEC) were downloaded from the NCBI Sequence Read Archive. The raw read counts per gene for all samples were calculated as described above and were normalized for library size by converting to CPM (counts per million) using edgeR [26]. The R package “sva” (v3.36.0) [27] was applied to adjust for batch effects, along with information of histological subtype of each sample. PCA was performed using the “prcomp” function in R.

### Variant calling

Single Nucleotide Polymorphism (SNP) discovery and filtering from RNA-seq data were performed using HaplotypeCaller under standard parameters according to GATK [28] (v4.1.8) Best Practices (https://github.com/gatk-workflows/gatk3-4-rnaseq-germline-snps-indels). Additionally, SNPs with a depth < 25 and an allele frequency < 0.2 were excluded. The functional effects of the mutations were predicted using SnpEff (v5.0) [29], and SNPs with "high" or "moderate" functional importance were retained. To visualize representative genes that are mutated in salivary gland tumors in COSMIC [30], the vcf format data was converted to maf format data using ANNOVAR [31] and annovarToMAF under standard parameters, and the "waterfall" function of R package GenVisR (v1.20.0) [32] was applied.

### Detection of fusion genes

Candidate fusion genes were deduced from RNA-seq data using STAR-Fusion (v1.6.0) [24], FusionCatcher (v1.20) [33], and a combination of kallisto (v0.46.2) [34] and pizzly (v0.37.3) [35]. The detected candidate fusion genes were cross-referenced to ChimerDB4.0 [36], and those reported in salivary gland carcinoma were extracted and validated by RT-PCR. RT-PCR was performed as previously described [18] using a PrimeScript 1st strand cDNA Synthesis Kit (TAKARA-BIO), and the resulting RT-PCR products were subjected to Sanger sequencing at Macrogen Japan (Tokyo, Japan). All primers used are listed in Supplementary Table S5.

### Mycoplasma detection

Organoids were routinely tested for Mycoplasma contamination using an e-Myco VALiD Mycoplasma PCR Detection Kit (iNtRON Biotechnology, Seoul, Korea). All experiments were performed with mycoplasma-free cells.

### Statistical analysis

Associations between the establishment rate of each experimental model and the clinical information of the patients were tested using Fisher's exact ratio test and Student’s *t*-test in the open-source R Statistical Computing software (http://www.r-project.org/). Statistical significance was set at *p* < 0.05.

## Results

### Establishment of SGC PDOs and PDX models

**Fig. 1:**
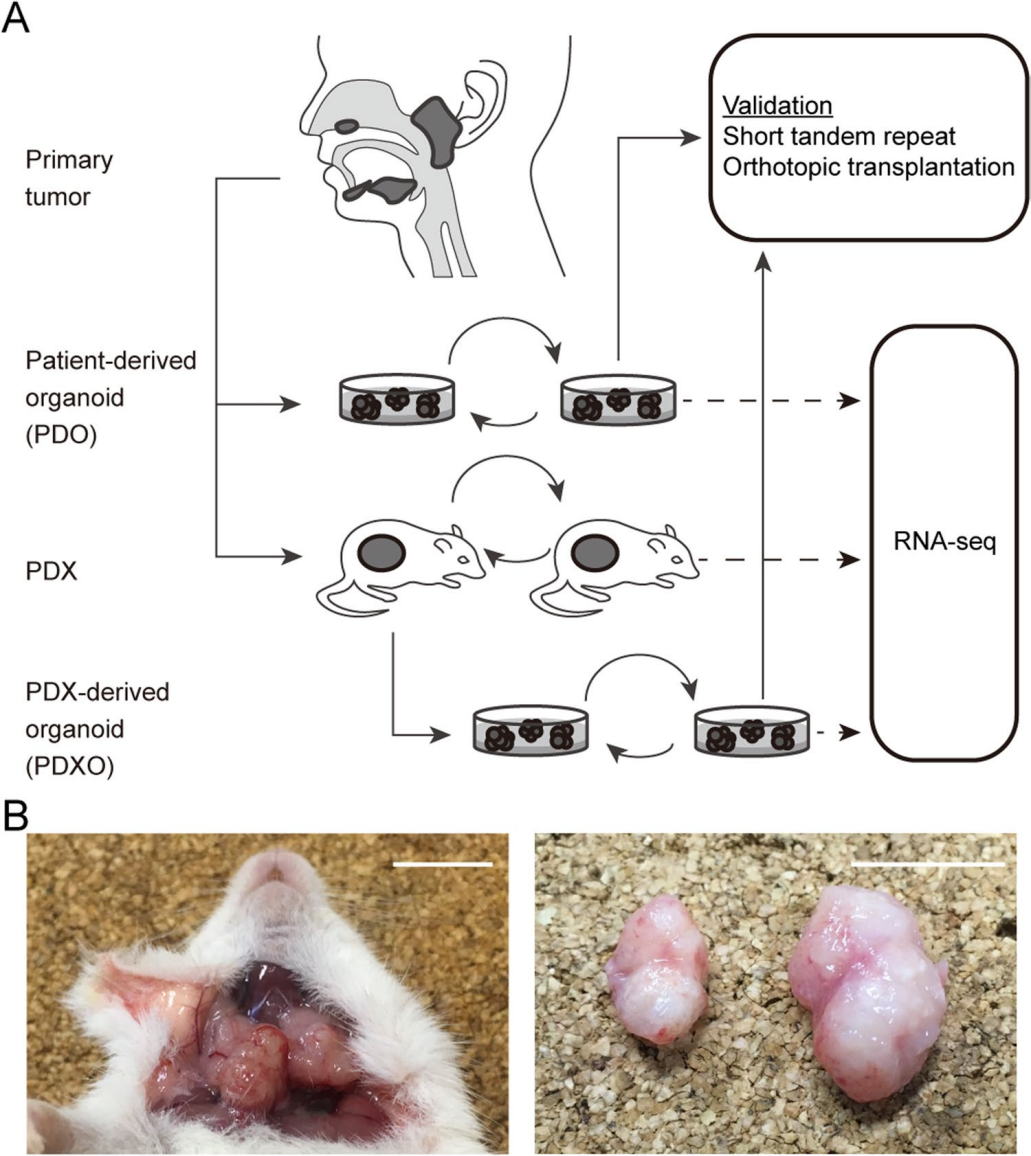
Establishment of salivary gland carcinoma (SGC) patient-derived xenografts (PDXs), organoids and orthotopic transplants. **A** Overview of the experiments. The patient specimens were divided into PDXs and/or organoid cultures. When PDXs were successfully established, we attempted to culture PDXOs. RNA-seq was performed on all established PDXs, patient-derived organoids and PDXOs, but could not be performed on the primary tumors due to insufficient sample tissue available. **B** Representative orthotopic transplants formed in the submandibular glands of mice. A PDXO from salivary duct carcinoma (YCU-SDC-20X) was transplanted into the left and right submandibular glands of a mouse and formed tumors, respectively. Scale bars, 10 mm

We established a series of SGC PDOs and PDX models using human SGC tumor sections of multiple histological subtypes (Table 1) by our existing protocol for human-ACC derived organoid and PDX models [18]. Comprehensive clinical information of the patients involved in the present study is shown in Supplementary Table S1. Based on the overview of our examinations (Fig. 1), we aimed to establish both SGC PDOs and PDXs when enough tumor specimens were secured. Additionally, we sought to generate PDXOs by ex vivo organoid culture of cells isolated from the established PDX tumors. PDX establishment was considered successful when two or more passages were possible, and organoid establishment was considered successful when five or more passages were possible and continuous growth was observed. Overall, we established PDXs from 6 (20.7%) of the 29 patients, whereas PDOs were established from 4 (11.4%) of the 35 patients (Supplementary Table S1, the data include PDXs and PDOs from ACCs reported previously). It has to be noted that no successful PDO cultures were established without coincident PDX establishment, except YCU-SDC-32. Additionally, PDXOs were generated from 3 (50.0%) of the 6 established PDXs (Supplementary Table S1).

**Table 1:** Patient characteristics. Summary of patient-derived xenograft (PDX), patient-derived organoid (PDO) and PDX-derived organoid (PDXO), corresponding clinical data

Name	Sex	Age	Pathological diagnosis	Primary site	TNM stage (7^th^ edition)			Last passage		
					T	N	M	PDX	Patient-derived organoid	PDX-derived organoid
YCU-ACC-1	F	48	Adenoid cystic carcinoma	Nasal cavity	4a	0	0	10^a^	2^b^	4^b^
YCU-ACC-4	M	67	Adenoid cystic carcinoma	Sublingual gland	4a	2c	1	10^a^	9^b^	9^b^
YCU-SDC-14	M	51	Salivary duct carcinoma	Submandibular gland	3	3b	0	8^a^	55^a^	35^a^
YCU-MYEC-16	M	73	Carcinoma ex pleomorphic adenoma (Myoepithelial carcinoma)	Parotid gland	2	0	0	7^a^	1^b^	5^b^
YCU-SDC-20	M	71	Salivary duct carcinoma	Parotid gland	4a	1	0	9^a^	52^a^	40^a^
YCU-MEC-24	F	55	Mucoepidermoid carcinoma	Oral floor	4a	2b	0	5^a^	25^b^	35^a^
YCU-SDC-32	M	72	Carcinoma ex pleomorphic adenoma (Salivary duct carcinoma)	Parotid gland	2	0	0	0^b^	36^a^	Not available

a As of March 2021

b Not being actively passaged at the time of publication

The clinical stage of the tumor was at the time of the first treatment, not during the entire clinical course of the patient. See Supplemental Table 1 for information on all patients from whom the specimens were collected

**Fig. 2:**
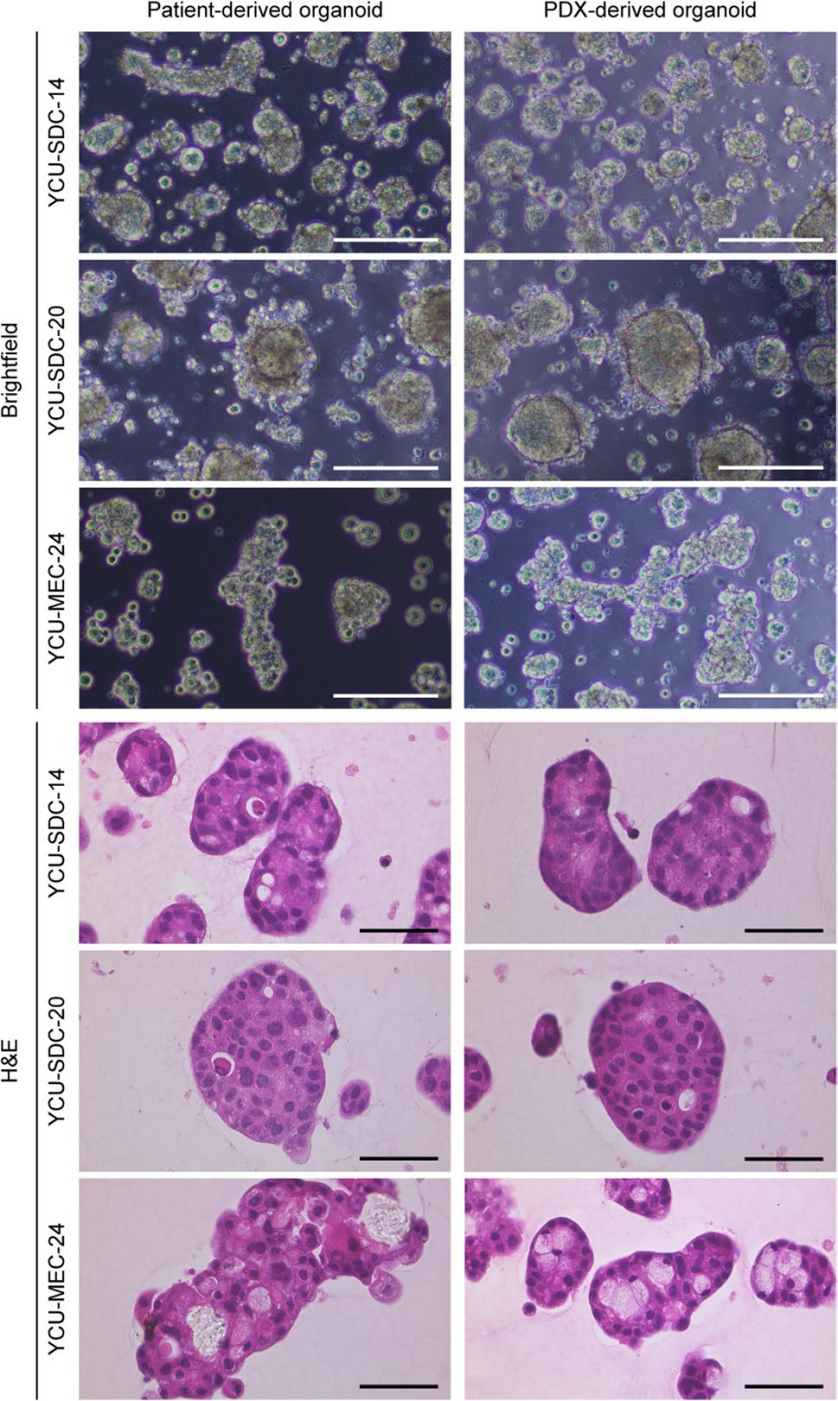
Brightfield images and hematoxylin and eosin (H&E) staining of salivary gland carcinoma (SGC) organoids. The left column shows the patient-derived organoids, and the right column shows the patient-derived xenograft (PDX)-derived organoids. The patient-derived organoids and PDXOs derived from salivary duct carcinoma (YCU-SDC-14 series, YCU-SDC-20 series) both showed cyst-like structures with necrosis inside. Those from mucoepidermoid carcinoma (YCU-MEC-24 series) both showed grape-like structures and glandular tuft formation. Scale bars represent 50 µm for brightfield images and 20 µm for H&E staining. See Fig. S1. for another salivary duct carcinoma organoid (YCU-SDC-32)

As yet, four PDOs have been successfully established from patients with SDC (YCU-SDC-14, YCU-SDC-20, and YCU-SDC-32) and MEC (YCU-MEC-24) in this study. In addition, PDXs have been established using SDC (YCU-SDC-14 PDX, YCU-SDC-20 PDX), MEC (YCU-MEC-24 PDX) and MYEC (YCU-MYEC-16 PDX). Additionally, PDXOs (YCU-SDC-14X, YCU-SDC-20X, and YCU-MEC-24X) have successfully been generated using YCU-SDC-14 PDX, YCU-SDC-20 PDX and YCU-MEC-24 PDX, respectively. The SGC PDOs and PDXOs showed similar characteristics when aggregated on Matrigel according to their origins (Fig. 2 and Supplementary Fig. S1). Each generated organoid was confirmed to have a genetic match with the original tumor by short tandem repeat (STR) profiling (Supplementary data). Thus, YCU-SDC-14, YCU-SDC-20 and YCU-MEC-24 specimens were suitable for the generation of PDOs, PDXs and PDXOs (Table 1).

Our SGC PDX models could be maintained up to 8 passages. The YCU-SDC-14 PDX model often developed liver metastases after the first passage (Supplementary Fig. S2A). SGC PDOs and PDXOs were cultured at a passaging ratio of 1:1.5 to 1:2 approximately every 14 days, with up to 55 passages. These organoids and PDXs showed a wide spectrum of proliferative activity, e.g. YCU-SDC-32 could be passaged more than 35 times as patient - derived organoid, whereas the growth of PDXs was slow, not yielding sufficient quantities for passaging PDX or PDXO cultures.

We did not observe any correlation between the establishment success rate for each model and clinical/histological characteristics (Supplementary Table S1). Our models could successfully be recovered after long-term preservation (at least 6 months at − 80 °C; Supplementary Fig. S3).

### PDXs and orthotopic mouse models from SGC organoids retain their original histological features after passaging

Next, we evaluated whether our SGC organoids and PDXs could recapitulate the histological characteristics of the original tumors. Most SGCs are usually well-differentiated tumors, resulting in difficult diagnoses by using only one specific histological marker. Thus, we deem it necessary to assess at a wide range of histological images to ensure an accurate diagnosis [3]. Since our in vitro organoids did not show sufficient histological structures, we set out to establish an orthotopic animal model from our PDOs and PDXOs, as described previously [18], followed by histological analysis of the orthotopically transplanted organoids. We observed palpable tumor formation 2–4 weeks after transplantation (Fig. 1B), and they required approximately six months to reach a 1 cm diameter.

**Fig. 3:**
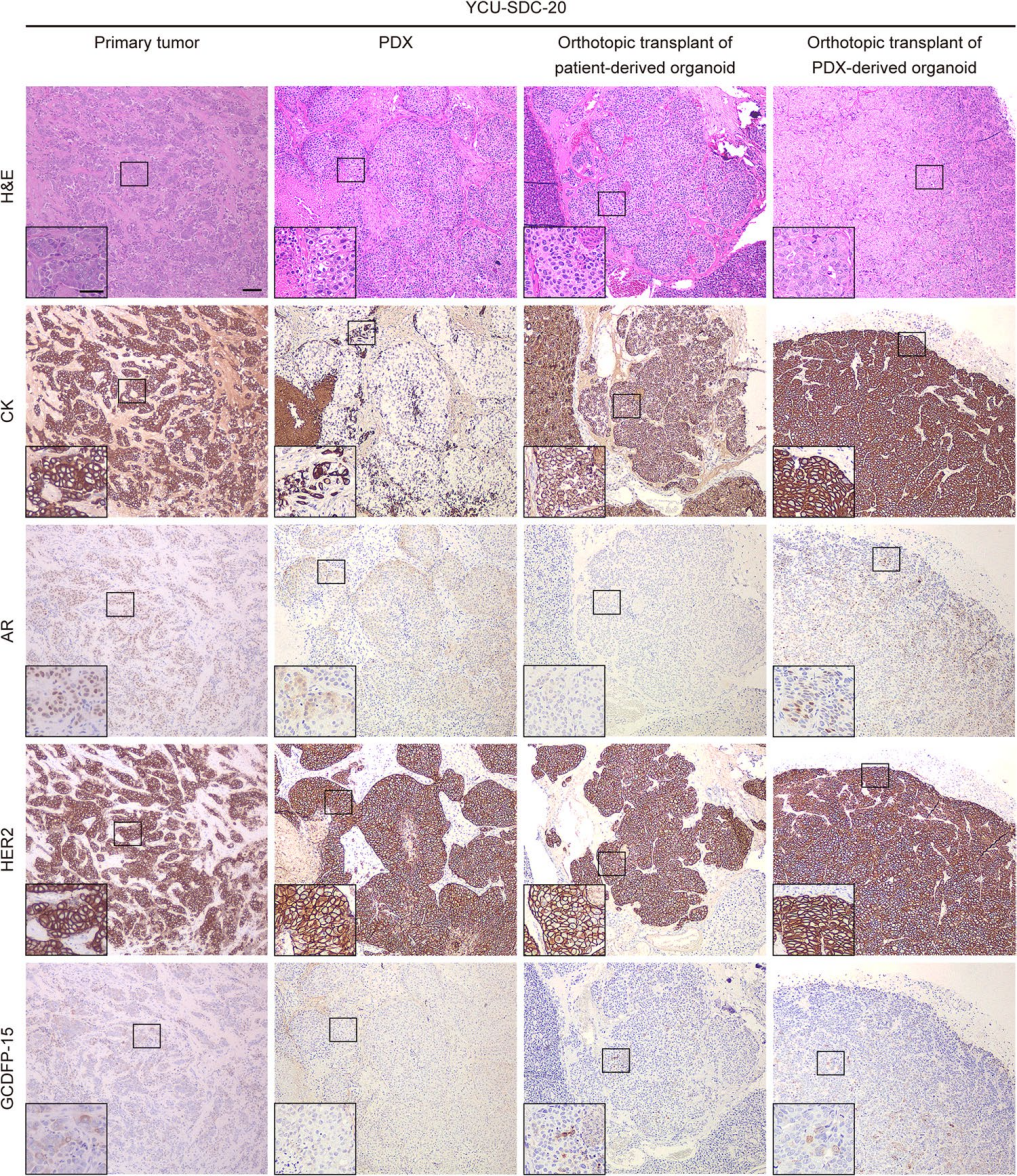
Histology and IHC staining. Hematoxylin and eosin (H&E) staining and IHC (CK, AR, HER2, GCDFP-15) of the patient’s primary tumor, patient-derived xenograft (PDX), orthotopically transplanted PDO, and orthotopically transplanted PDXO. A case of salivary duct carcinoma (YCU-SDC-20 series) is presented as a representative. For other cases, see Fig. S2A-G. Scale bars in a large frame represent 10 µm, and those in a small frame represent 5 µm

Histologically, the PDXs and orthotopic transplants from PDOs and PDXOs showed morphologies similar to the originating SGC tumors, as confirmed by independent pathologic assessment (Fig. 3 and Supplementary Fig. S2A–G). Highly differentiated histologic characteristics, such as cribriform structures with comedonecrosis in SDC (YCU-SDC-14 and YCU-SDC-20) and cystic structures lined by mucous cells and clear cells in MEC (YCU-MEC-24), were present in both PDXs and orthotopic transplants, similar to the matched patient samples. PDXs of YCU-MYEC-16 also recapitulated the highly differentiated characteristic of MYEC, showing exclusive myoepithelial differentiation and clear-cut tumor cell infiltration into adjacent salivary glands. Using IHC for CK as an epithelial marker and p63 as a myoepithelial marker, we observed features similar to those in the matched patient samples. Furthermore, we found that PDXs and orthotopic transplants from PDOs and PDXOs retained expressed of the human epidermal growth factor receptor 2 (HER2), which is frequently seen and may serve as a potential therapeutic target in SDC. Expression of the androgen receptor (AR) in the original tumor of YCU-SDC-14 was not retained after passaging in PDXs or orthotopically transplanted organoids. AR and GCDFP15 expression in the original tumor of YCU-SDC-20 was not confirmed in PDXs and orthotopically transplanted PDOs, but their expression was retained in orthotopically transplanted PDXOs. Overall, we found that all established organoids had the potential to generate orthotopic transplants and that these organoids and PDXs recapitulate the histological characteristics of the original tumors.

### Transcription profiles of PDXs, PDOs and PDXOs of SGC

Next, all established PDXs and organoids were genetically characterized on the basis of their transcription profiles as determined by RNA-seq analysis. The original tumors could not be characterized due to lack of tissue. To account for the possibility of murine stromal cell contamination in PDXs or PDXOs, we performed bioinformatics analysis to distinguish between human and mouse-derived reads before estimating gene expression levels (Supplementary Fig. S4). Only human-derived reads were used for analyzing the transcription profiles.

**Fig. 4:**
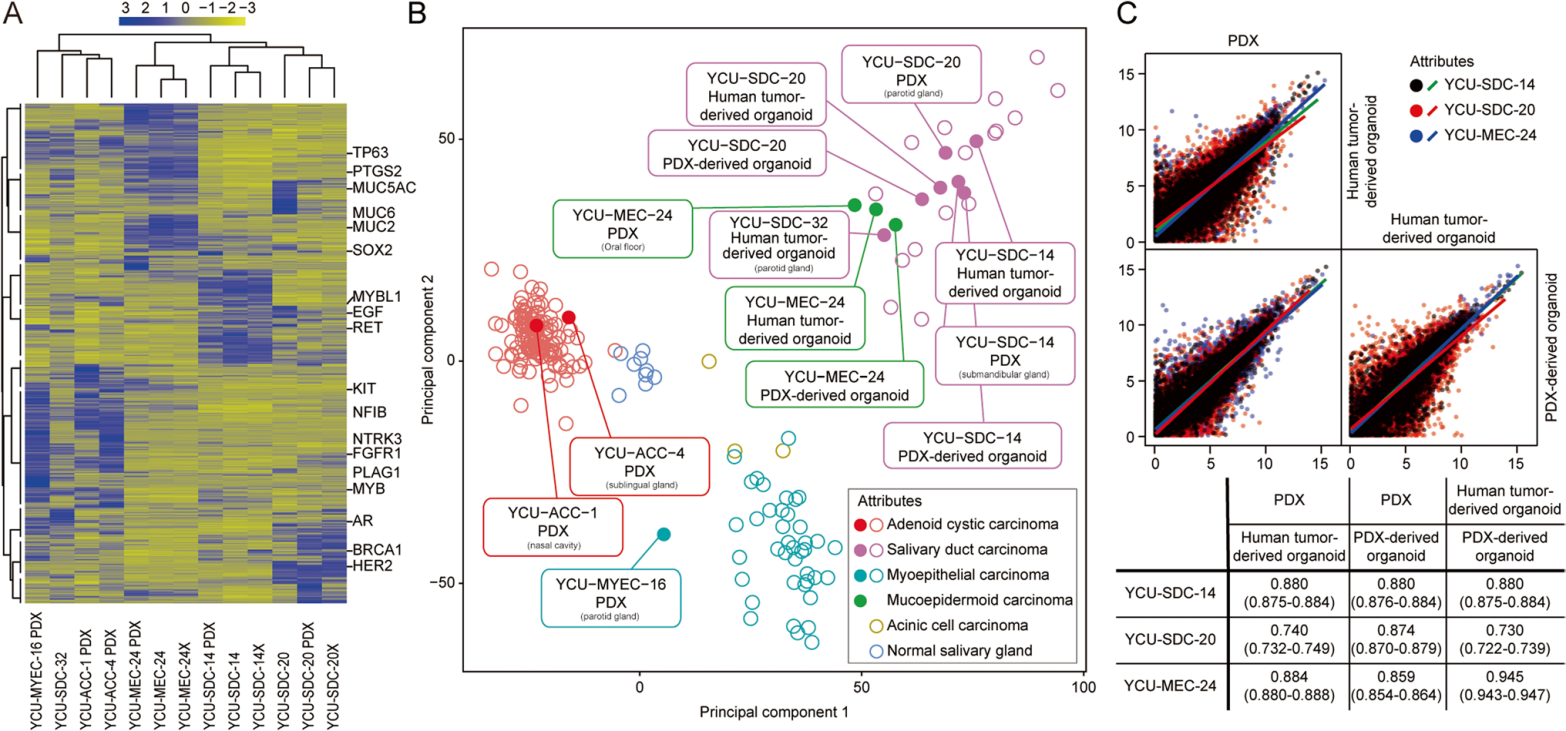
Gene expression analysis. **A** Heatmap showing the top 2000 variable genes for patient-derived xenografts (PDXs) and organoids. **B** PCA plot. The filled circles indicate the established PDX and organoid samples. Blank circles indicate gene expression data of salivary gland carcinomas (SGCs) with multiple histological subtypes or normal parotid tissues downloaded from public databases. All data were corrected for batch effects based on the tissue type. **C** Correlation matrix. Gene expression levels scatter plots for each experimental model were plotted for patient samples in which the patient-derived organoids, PDXs and PDXOs were established (YCU-SDC-14, YCU-SDC-20, and YCU-MEC-24). The Pearson correlation coefficient and 95% confidence interval are shown in the table below

After heatmaps were obtained based on the estimated gene expression levels, each model was hierarchically clustered according to patient origin and histological subtype (Fig. 4A). In addition, these results were combined with gene expression profiles of 180 cases of SGC, including multiple histological subtypes obtained from public databases, which confirmed that our PDXs and organoids were clearly classified into each SGC category (Fig. 4B). Next, correlation coefficients were calculated between models having the same origin to quantify the similarity of these expression profiles. We found that the gene expression levels across models having the same origin were highly correlated with a mean Pearson correlation of 0.834, PDXs vs. PDOs, a mean Pearson correlation of 0.871, PDXs vs. PDXOs and a mean Pearson correlation of 0.851, PDOs vs. PDXOs (Fig. 4C).

Next, we explored the presence of fusion genes using RNA-seq data. In addition to the previously reported *MYBL1-NFIB* fusion gene in YCU-ACC-4, the frequently reported *CRCT1-MAML2* fusion gene was detected in all PDXs, PDOs, and PDXOs of YCU-MEC-24. Moreover, its presence was reconfirmed using Sanger sequencing (Supplementary Fig. S5). No other fusion genes were detected in our models.

### Genomic variation in PDXs, PDOs and PDXOs of SGC

**Fig. 5:**
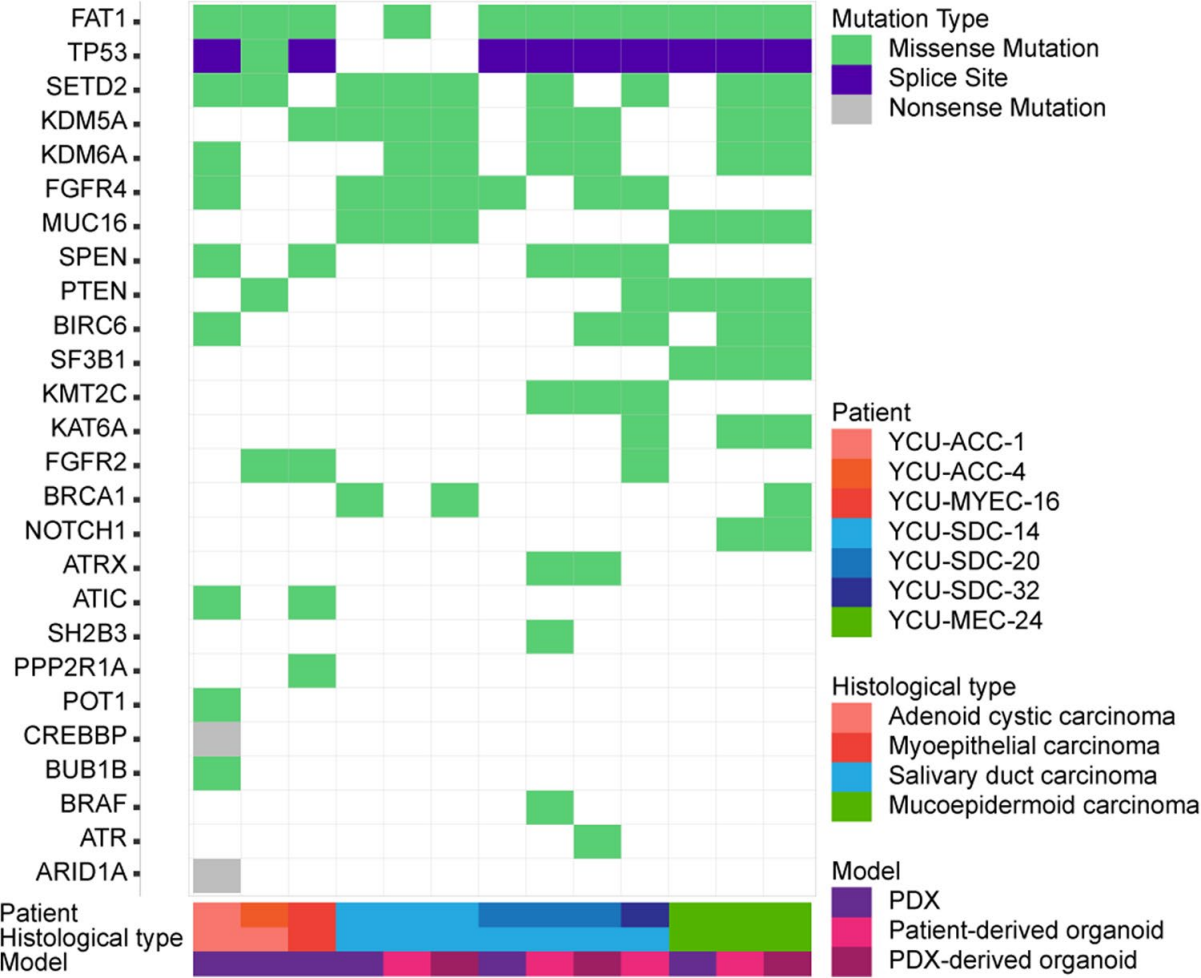
Gene mutation/variant analysis. Genetic variants detected in RNA-seq data of each established model Genetic variants were annotated, and typical possible effects on genes are shown. See Supplementary Table S2 for a list of all genetic variants

Since we did not have access to sufficient primary tumor tissues or patient blood samples for genome sequencing, we performed a limited analysis of genomic variation in the established models using RNA-seq data. The SNP and indel outputs according to GATK [28] Best Practice (https://software.broadinstitute.org/gatk/best-practices/) were filtered using the COSMIC database [30] (https://cancer.sanger.ac.uk/cosmic). All extracted mutations are listed in Supplementary Table S2. Among these results, genomic mutations frequently found in SGCs are also shown in Fig. 5. *TP53* mutations, frequently observed in SGC [11], were detected in all samples other than those derived from YCU-SDC-14. In contrast, *PIK3CA* mutations, which have been reported in SDC and ACC [11], were not detected in our series.

## Discussion

We report the generation of in vitro and in vivo models of multiple SGC histological subtypes using our previously established approach for organoid and PDX culture [18]. We confirmed both a histological and a genetic reproducibility of our all PDXs, PDOs and PDXOs established in this study. Based on these results, we conclude that our approach may be used to overcome the problem of a lack of pre-clinical SGC model systems due to its scarcity and slow-growing characteristics.

To date, several studies have reported the establishment of SGC cell lines [37–42]. As yet, MDA-SDC-04 is the only SDC cell line established using a 2-dimensional culture system [40], but the authors reported that this cell line requires an immortalization process and loses chromosomal aberrations by long-term passaging without any tumor-forming potential in xenografts. Here, we report for the first time the establishment of PDXs and organoids originating from human SDC tumors with histological similarities to the original tumors by orthotopic transplantation of SDC organoids. Their genetic reproducibility as SDC models could be confirmed by RNA-seq analysis. While the establishment of most previously reported SGC cell lines was based on only a single histological subtype each with a very limited number of lines, our method allowed the establishment of several in vitro and in vivo models of various histological subtypes. Our results underscore the notion that organoid culture can be applied to a number of malignancies and may serve as an approach to preserve more of the original tumor characteristics than traditional 2-dimension culture techniques. We should mention, however, that our establishment rates of organoids and PDXs were lower than those obtained for other malignancies such as breast or lung cancers. Interestingly, we observed different SGC model establishment rates and different morphologies of PDOs/PDXOs even within the same histology type such as SDC. We did not observe any correlation between the establishment success rate for each model/organoid morphology and histological characteristics (Supplementary Table S1). We additionally performed gene expression analyses between YCU-SDC-14 PDO/YCU-SDC-20 (establishing PDX, PDO and PDXO) and YCU-SDC-32 PDO (establishing only PDO) using RNA-seq data (Supplementary Fig. S6). Although it seems that there is a difference in the principle component analyses (PCAs) between YCU-SDC-14 PDO/YCU-SDC-20 and YCU-SDC-32 PDO, it is difficult to draw firm conclusions due to the lack of biological replicates. The histological/genetic differences between SGC models affecting model establishment or the morphology thereof, may become clearer in the future when the number of successful models that have been established increases.

Our SGC PDX establishment rate was lower than that reported previously [43]. This observation might be attributed to the different histological subtypes of SGCs included in the studies, the amount of SGC tissue collected from surgical resections and/or the features of the acinar/ductal epithelia phenotype in primary resection sites. The parotid gland consists predominantly of serous glandular epithelium, whereas the sublingual and minor salivary glands differ in histological and parenchymal structure due to their different fetal development. In fact, our success rates of PDX establishment from parotid/submandibular and sublingual/minor salivary glands were 17.4% and 33.3%, respectively, indicating that the establishment of SGC models may vary depending on the tissue of origin. In addition, it should be noted that PDX models may behave inconsistently through passage and that PDXs may increase their growth rate with time [44]. We found that the doubling time of most SGC PDXs tended to gradually decrease through passage (Supplementary Fig. S7). Furthermore, since we only presented the mutational status of the successfully grown cultures, we cannot exclude the possibility that a specific set of mutation(s) may predispose SGCs to be successfully grown as PDXs and/or organoids, although we have no information on which genetic changes might be associated with it. Further analyses, such as spatial proteogenomics analysis, may provide useful information to resolve this issue.

We also found that PDX tumors may generate organoids (PDXOs) that are homologous to PDOs for SDC and MEC, as we previously reported for ACC [18]. Particularly, we found that our SDC PDXOs showed similar gross cyst formation features and histological properties of the orthotopic transplants with similarities in gene expression. These results are in conformity with previously reported methods for PDXOs of pediatric liver cancer [45] and non-small lung cancer [46].

Our SGC organoids proliferated very slowly as do their primary tumors in some tissue types; therefore, the number of cells obtained from the culture process is limited. As the growth of PDO of YCU-MEC-24 was terminated during passage, the number of cells obtained from PDOs alone might be insufficient to use continuously for a variety of studies from both practical and cost perspectives. In such a case, the use of PDXO organoids is thought to be an alternative method that can overcome this issue concerning the culture of slow-growing cancers, since we confirmed that PDX was capable of multiple passages, up to a maximum of approximately eight times, while securing tumor volume as well as maintaining the model without loss. As recently reported by Yoon et al., some modification of medium supplements, such as reducing the level of a potent anaplastic lymphoma kinase inhibitor, could affect the establishment rate or the growth of SGC organoids[47].

PDXs may potentially contain mouse mesenchymal cells [15] and hence, PDXO cultures carry the risk of mouse cell contamination. In fact, the PDXO of YCU-SDC-20 contained a relatively large number of mouse-derived reads and the PCR results for mouse-derived mitochondrial DNA sequences were positive (Supplementary Fig. S3), suggesting that the PDXO of YCU-SDC-20 may contain mouse cells. While we did not observe any significant differences in tumorigenic, histological and genetic profiles by orthotopic transplantation between our models in the present study, these results may be affected by the proportion of mouse cells. Therefore, it is necessary to always consider the risk of contamination when conducting research using PDX-related approaches.

Another limitation of the present study is that it did not fully reflect the highly differentiated and heterogeneous nature of SGC. First, we observed small differences in protein expression patterns by IHC of PDXs and orthotopically transplanted organoids. Although the primary tumor of YCU-SDC-14 was partially positive for AR, all our PDXs, PDOs and PDXOs of YCU-SDC-14 were AR negative. In addition, in the case of YCU-SDC-20 with an AR positive primary tumor, we found that the transcription profiles of YCU-SDC-20 and YCU-SDC-20 PDX/YCU-SDC-20X did not correlate well, while those of YCU-SDC-20 PDX and YCU-SDC-20X correlated very well. However, the PDXs and PDOs of YCU-SDC-20 were AR negative, whereas the PDX-derived tumors were AR positive. These phenomena may be due to the tumor heterogeneity [48] and/or clonal selection. Cancer cells may evolve and/or selectively change their properties through model establishment and passaging [44]. Therefore, the major AR expressing population may have become de-differentiated through passage in this study. Although our SDC models were not supplemented with testosterone, it is possible that testosterone supplementation could have maintained AR expression, as has been shown in prostate cancer PDX models [49]. When comparing the heterogeneity of primary SGCs and of our pre-clinical models, a major limitation of the current study is that we did not directly compare the reproducibility of gene expressions or gene mutations because we did not collect sufficient amounts of the primary patient tumors or other patient samples such as blood. Therefore, the retention of gene expression or mutation profiles through model establishment and its passaging was not explored in detail. Furthermore, normal salivary tissue of corresponding patients could be employed for further analysis such as proteomics to provide more insight into the progression of SGCs.

Despite these limitations, our results indicate that SGC related organoids from a variety of histological types may be used for the future development of novel therapies. In fact, we found that our SGC models are amenable for pharmacologic examinations in vitro as well as in vivo (data not shown). While the lack of in vitro and in vivo SGC models that recapitulate the diversity of human SGC has hampered our understanding of its pathogenesis and therapy response, our approach may overcome these limitations. In the future, our approach may be expanded to more malignancies and histological subtypes with higher establishment rates by using new culture techniques such as conditional reprogramming [50].

In conclusion, we successfully generated PDXs and PDOs as in vitro and in vivo models of SDC, MEC and MYEC, in addition to ACC. Additionally, we show that PDX tumors can be used to derive SDC and MEC organoids. We confirmed that our established PDXs, PDOs and PDXOs retain their original histological and genetical features throughout passaging. The framework of our developed organoids and PDX-related SGC models shows potential for application in preclinical studies aimed at the development of novel treatment modalities for patients diagnosed with rare cancers, including SGC, and may be used for elucidating the molecular biology underlying these diseases.

## Supplementary Information

Below is the link to the electronic supplementary material.

Supplementary file1 (DOCX 7346 KB)

Supplementary file2 (XLSX 19 KB)

Supplementary file3 (XLSX 81 KB)

Supplementary file4 (XLSX 28 KB)

Supplementary file5 (XLSX 913 KB)

Supplementary file6 (CSV 89 KB)

Supplementary file7 (PDF 2227 KB)

## Data Availability

The RNA sequencing data that support the findings of this study have been deposited in the DNA Data Bank of Japan Sequence Read Archive (DRA) under accession number DRA011243. All data supporting the findings of the study are available within the article and its Supplementary Information or are available from the corresponding author upon request.

## Abbreviations

*ACC*: Adenoid cystic carcinoma
*DMEM*: Dulbecco's Modified Eagle Medium
*DNA*: Deoxyribonucleic acid
*GFR*: Growth-factor-reduced
*IHC*: Immunohistochemistry
*NSG*: NOD Cg‐*Prkdc* ^scid^ *Il2rg* ^tm1Wjl^
*MEC*: Mucoepidermoid carcinoma
*MYEC*: Myoepithelial carcinoma
*PDX*: Patient-derived tumor xenograft
*RNA*: Ribonucleic acid
*PCA*: Principal component analysis
*PDO*: Patient-derived organoid
*PDXO*: PDX-derived organoid
*RT-PCR*: Reverse transcription polymerase chain reaction
*SGC*: Salivary gland carcinoma
*STR*: Short tandem repeat
