# Supplementary material for: Establishment of experimental salivary gland cancer models using organoid culture and patient-derived xenografting
Source: Cell Oncol (Dordr). 2022 Dec 20;46(2):409–21. doi: 10.1007/s13402-022-00758-6 (PMC10060313; doi:10.1007/s13402-022-00758-6)
Supplement: Supplementary file 1 — Supplementary file1 (DOCX 7346 KB) [file 13402_2022_758_MOESM1_ESM.docx]

**Experimental models of salivary gland cancer established using organoid culture and patient-derived xenografting**

Yoshihiro Aizawa, Kentaro Takada, Jun Aoyama, Daisuke Sano, Shoji Yamanaka, Masahide Seki, Yuta Kuze, Jordan Ramilowski, Ryo Okuda, Yasuharu Ueno, Yusuke Nojima, Yoshiaki Inayama, Hiromitsu Hatakeyama, Takashi Hatano, Hideaki Takahashi, Goshi Nishimura, Satoshi Fujii, Yutaka Suzuki, Hideki Taniguchi, Nobuhiko Oridate

**Supplementary Data**

- Supplementary Fig. S1-S7
- Supplementary Table S5


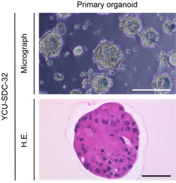


**Supplementary Fig. S1. Bright-field images and hematoxylin and eosin (H&E) staining of YCU-SDC-32**

Similar to the other two salivary gland carcinoma (SDC)-derived organoids, the primary organoids derived from YCU-SDC-32 showed a cyst-like structure. Scale bars represent 50 µm for bright-field images and 20 µm for H&E staining.


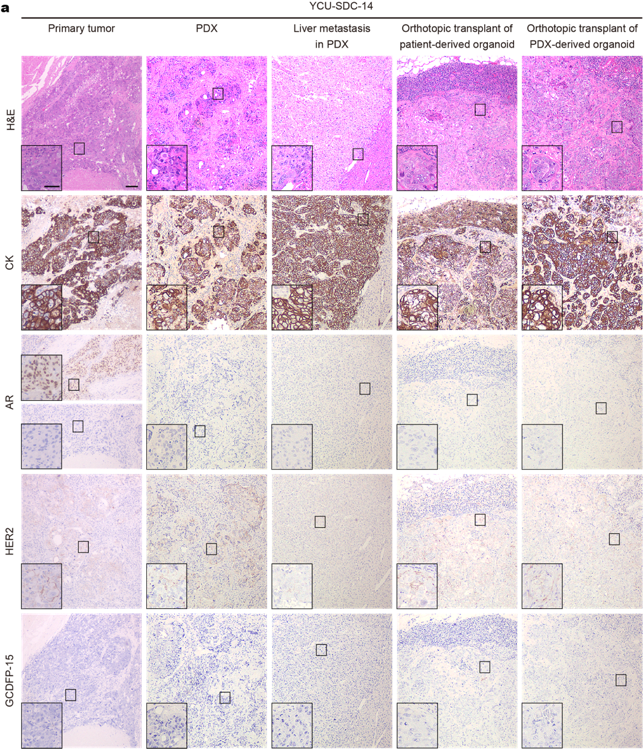


**
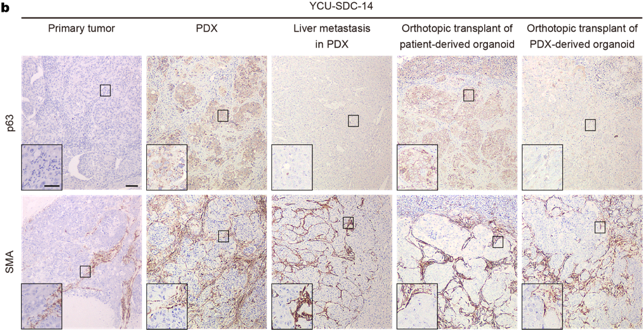
**

**
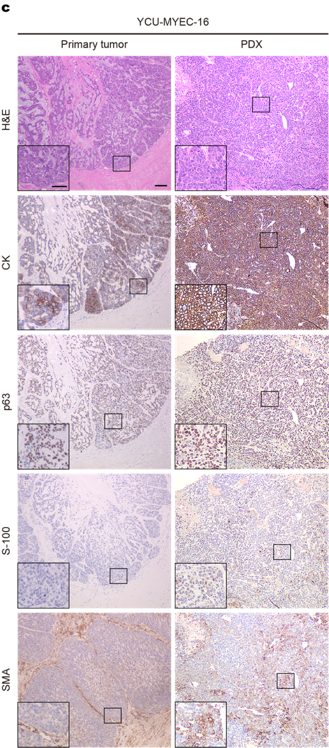
**

**
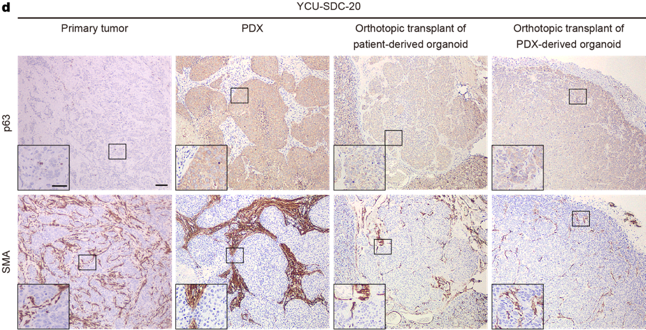
**

**
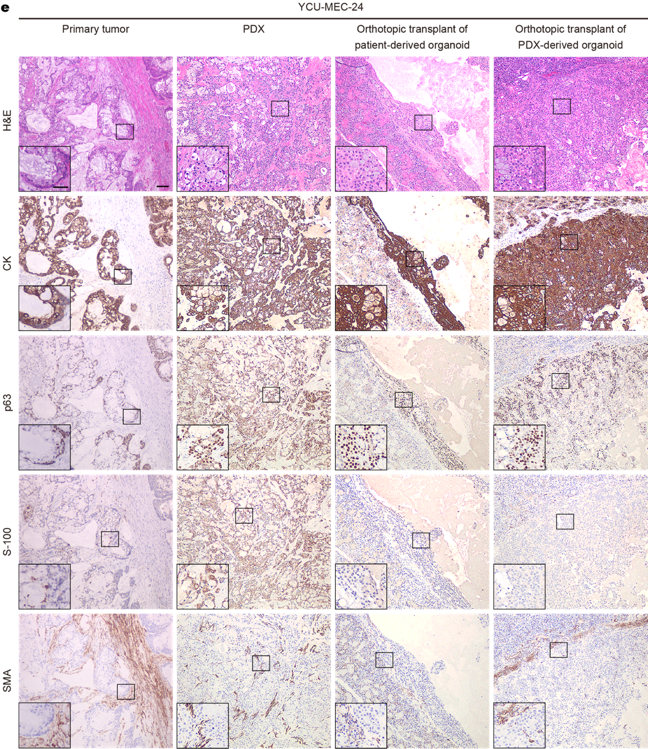
**

**
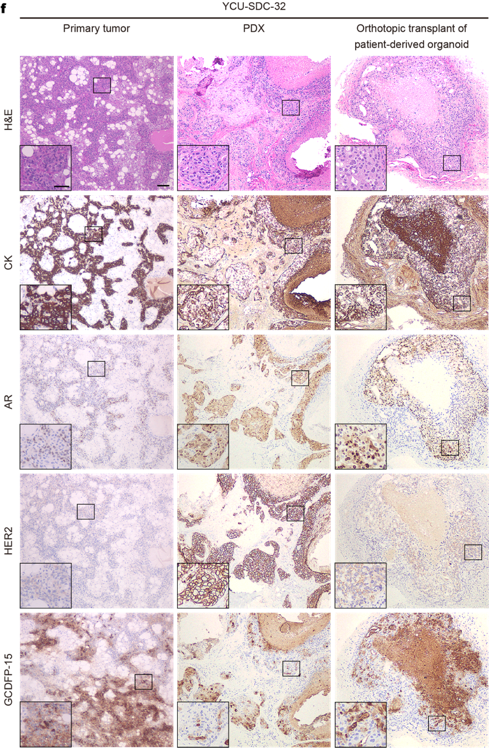
**

**
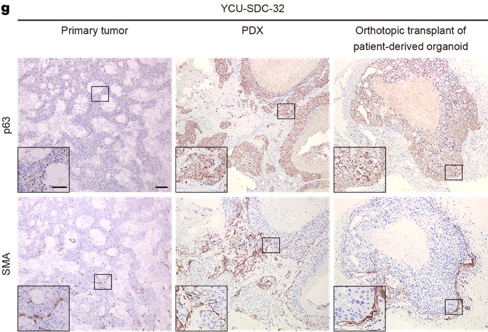
**

**Supplementary Fig. S2. Histology and IHC staining**

**a-b** YCU-SDC-14 series. **c** YCU-MYEC-16 series. **d** YCU-SDC-20 series. **e** YCU-MEC-24 series. **f-g** YCU-SDC-32 series. The primary tumor of YCU-SDC-14 had both AR-positive and AR-negative areas. Scale bars in a large frame represent 10 µm, and those in a small frame represent 5 µm.


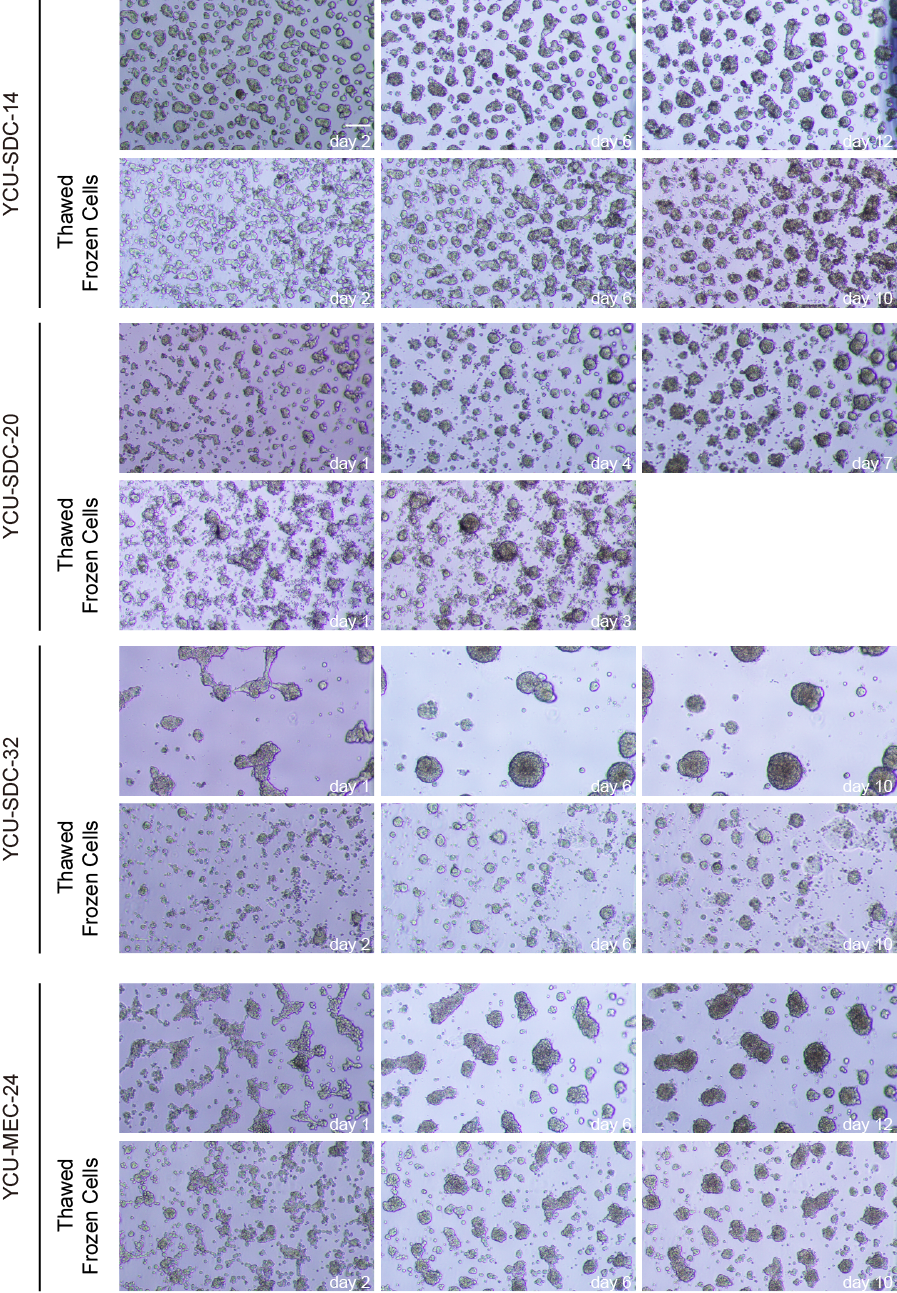


**Supplementary Fig. S3. Brightfield images of salivary gland carcinoma (SGC) organoids.**

The upper column shows the SGC organoids growing, and the lower column shows the SGC organoids growing after the long-term preservation for at least 6 months at −80°C.


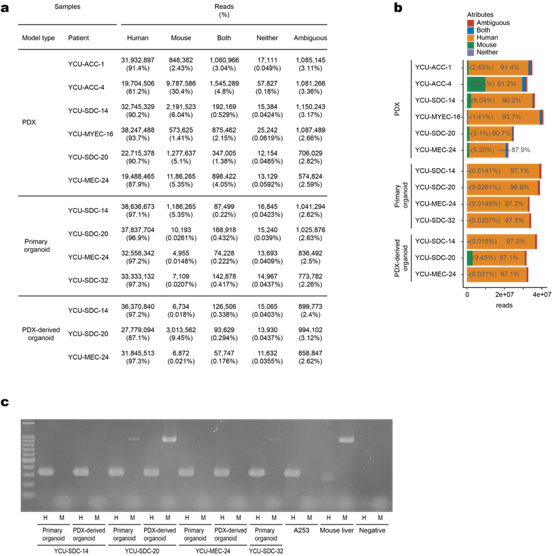


**Supplementary Fig. S4. Contamination of mouse cells in each patient-derived xenograft (PDX) and organoid**

**a** Distinguishing human and mouse reads from RNA-seq data using xenome. **b** is summarized in the bar graph. **c** Identification of animal species by PCR of mitochondrial DNA sequences. A253 cells were used as a positive control in humans, and mouse livers were used as a positive control in mice. The PDX-derived organoid of YCU-SDC-20 was positive in both humans and mice, indicating a mixture of both cell types.


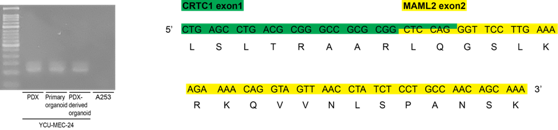


**Supplementary Fig. S5. Sangar sequence of *CRTC1-MAML2* fusion gene in YCU-MEC-24**


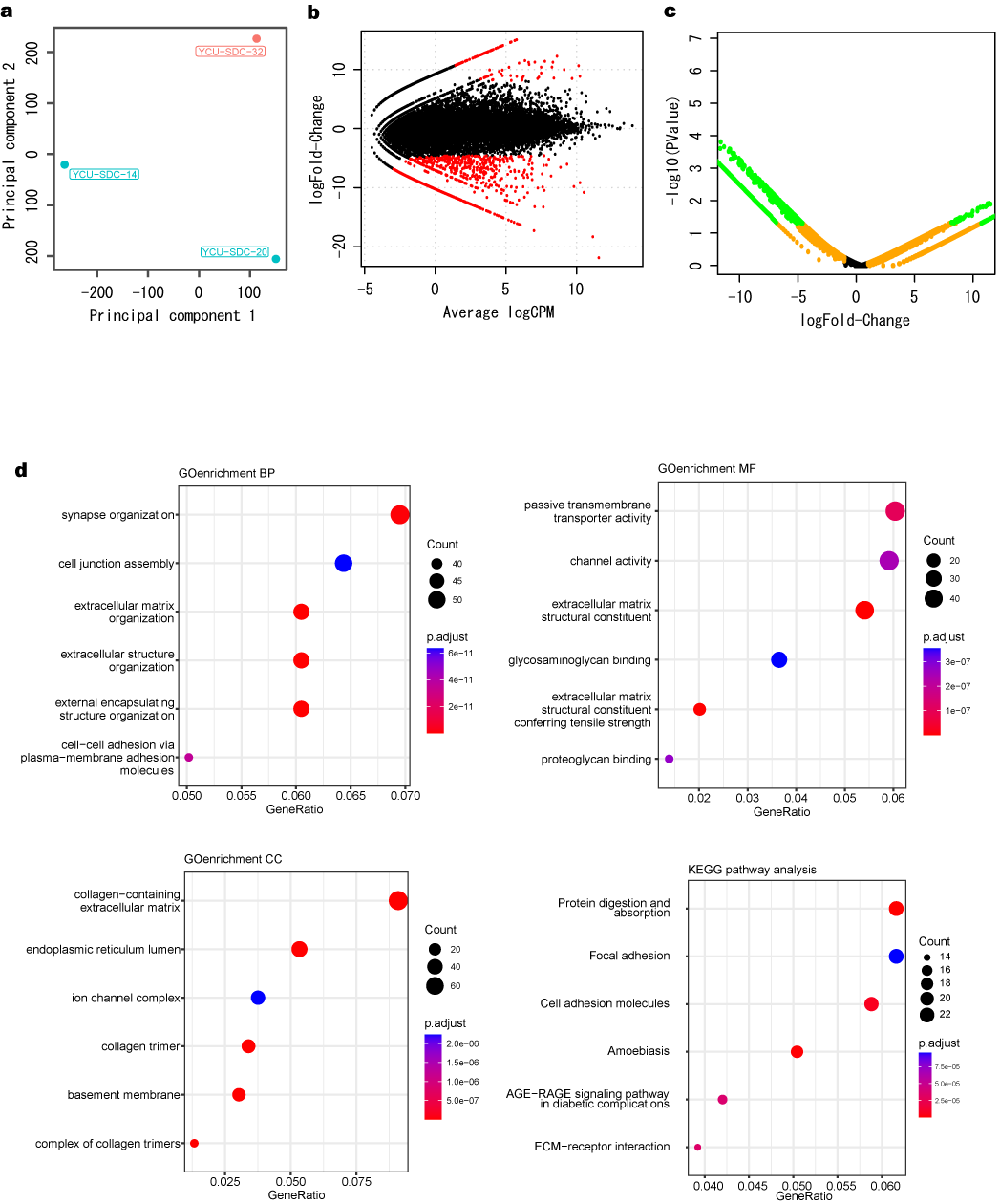


**Supplementary Fig. S6. Gene expression analysis between YCU-SDC-14 PDO/YCU-SDC-20 and YCU-SDC-32 PDO using RNA-seq**

**a** PCA plot. **b** MA plot. **c** Volcano plot. **d** Resuts of GO analyses and KEGG pathway analysis.

This gene expression analysis were perfomed as described in the main text. Briefly, RNA-seq reads were quality checked and adapter trimmed using fastp (v0.20.1) and the trimmed reads into those of humans (GRCh38/hg38) or mice (GRCm38/mm10) using xenome (v1.0.0) were distinguished to read only human reads for subsequent processing. The human reads were aligned to human genome reference sequence (GRCh38/hg38) using STAR (v2.7.5c) and counted for each gene using featureCounts (v2.0.1). The RNA-seq coverage and quality statistics are summarized in Supplementary Table S6. For differential gene expression (DEGs) analysis, the raw read counts per gene were then TMM normalized using edgeR (v3.30.3). Since the group (YCU-SDC-32 PDO) that did not establish PDX model lacked biological replicate, GLMCommonDisp function with options "robust=TRUE, subset=NULL, method=deviance" was used, followed by exactTest function. We regarded DEGs with a Benjamini-Hochberg adjusted p-value <= 0.05 and an absolute value of log2(fold change) >= 1 as statistically significant. We finally attempted to investigate the gene ontology terms and KEGG pathways that were related to SDC PDX establishment using R package “clusterProfiler” (v4.4.4) *.*


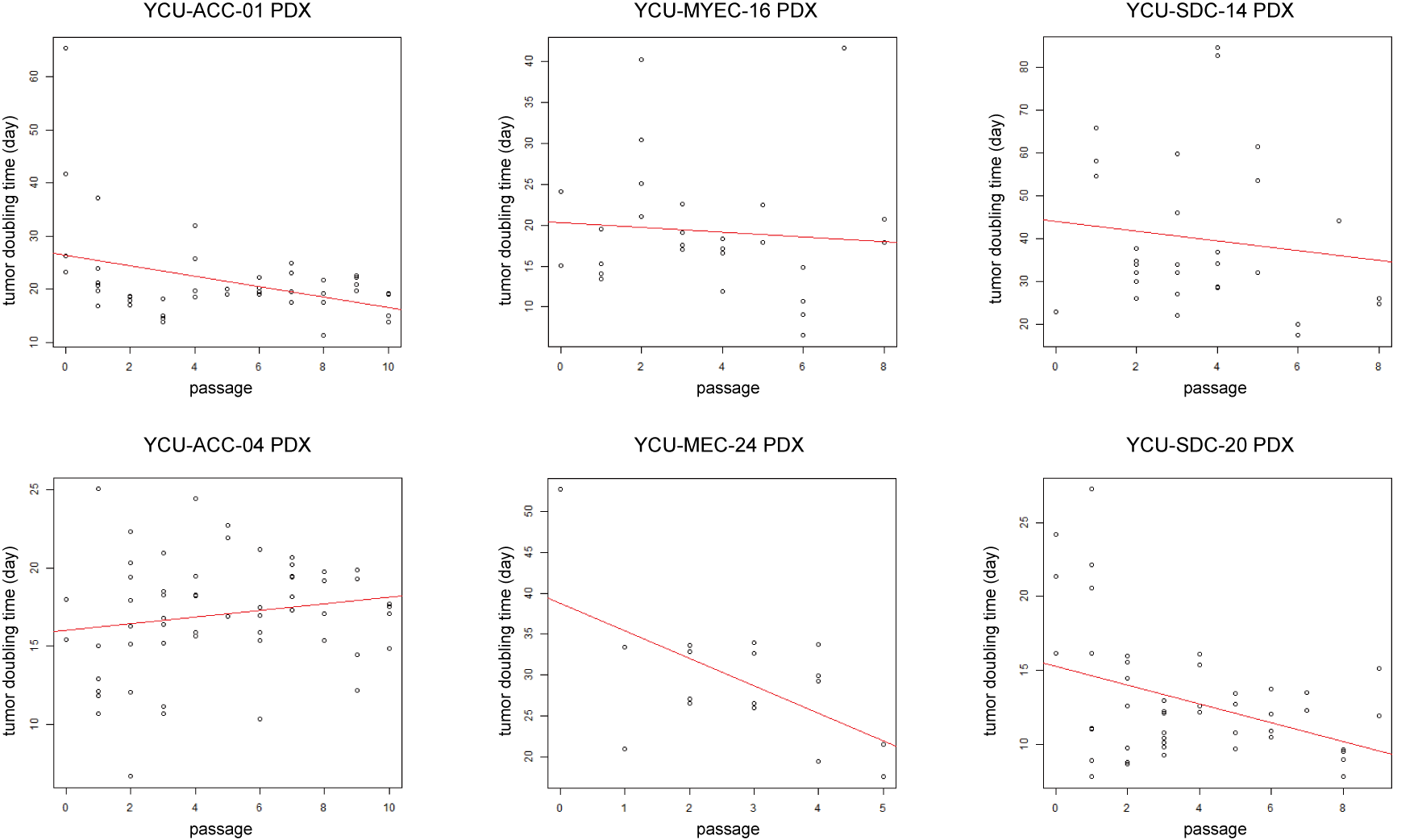


**Supplementary Fig. S7. Tumor doubling time of SGC PDX models.**

PDX tumor volume (mm^3^) was caluculated as long diameter (mm) × short diameter (mm) × short diameter (mm) × 0.5. Tumor doubling time was then obtained from linear regression via least squares method. Linear regression (red line) was shown using plotted tumo doubling time each passage.

| **Name** | **Forward / Reverse** | **Primer sequence (5’-3’)** | **Usage** |
| --- | --- | --- | --- |
| **CRTC1_outer** | Forward | AAGATCGCGCTGCACAATCA | RT-PCR, nested PCR  (Detection of fusion gene) |
| **CRTC1_inner** | Forward | GGAGGAGACGGCGGCCTTCG |  |
| **MAML2_outer** | Reverse | GGTCGCTTGCTGTTGGCAGG |  |
| **MAML2_inner** | Reverse | TTGCTGTTGGCAGGAGATAG |  |
| **S31_Human_F2** | Forward | CTCCTATTCTTGCACGAAAC | Genomic PCR  (Identification of species) |
| **S53_Human_R7** | Reverse | GATGGGGATTATTGCTAGGATG |  |
| **S07_Mouse_F** | Forward | GCACTGAAAATGCTTAGATGGATAATTG |  |
| **S08_Mouse_R** | Reverse | CCTCTCATAAACGGATGTCTAG |  |

**Supplementary Table S5. Primer information list**
