# Supplementary material for: Establishment of experimental salivary gland cancer models using organoid culture and patient-derived xenografting
Source: Cell Oncol (Dordr). 2022 Dec 20;46(2):409–21. doi: 10.1007/s13402-022-00758-6 (PMC10060313; doi:10.1007/s13402-022-00758-6)
Supplement: Supplementary file 7 — Supplementary file7 (PDF 2227 KB) [file 13402_2022_758_MOESM7_ESM.pdf]

Applied Biosystems
GeneMapper Software 5

201008

Sample File	Sample Name	Panel	OS	SQ
YCU-MEC-24_primary_tumor_D11_007.fsa	YCU-MEC-24_primary_tumor	GenePrint_10_v1.1		

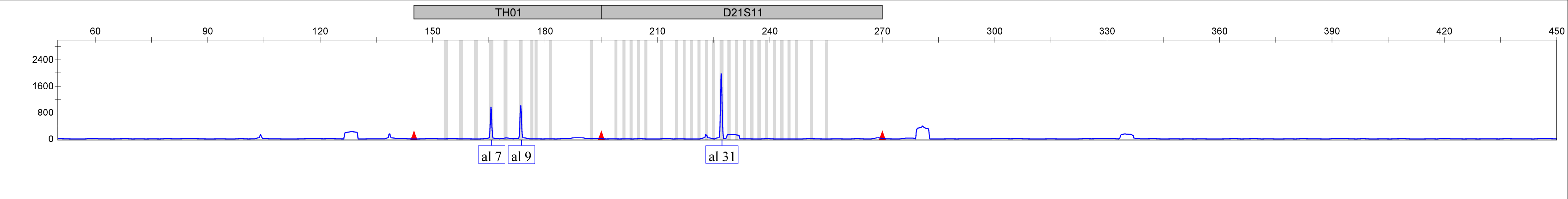

Sample File	Sample Name	Panel	OS	SQ
YCU-MEC-24_primary_tumor_D11_007.fsa	YCU-MEC-24_primary_tumor	GenePrint_10_v1.1		

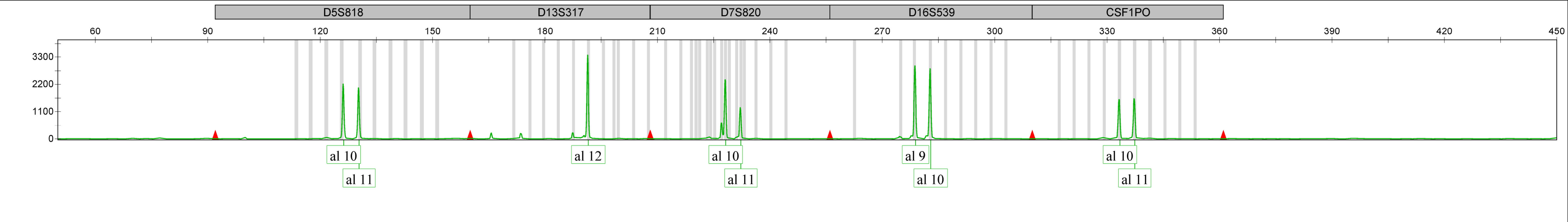

Sample File	Sample Name	Panel	OS	SQ
YCU-MEC-24_primary_tumor_D11_007.fsa	YCU-MEC-24_primary_tumor	GenePrint_10_v1.1		

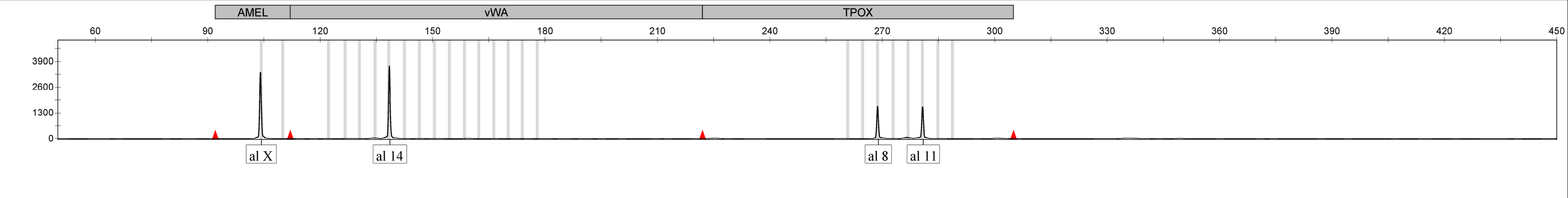

Applied Biosystems
GeneMapper Software 5

201008

Sample File	Sample Name	Panel	OS	SQ
YCU-MEC-24_H11_015.fsa	YCU-MEC-24	GenePrint_10_v1.1		

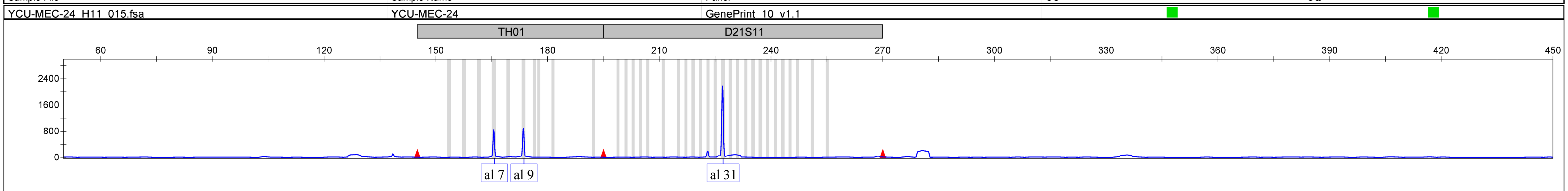

YCU-MEC-24_H11_015.fsa	YCU-MEC-24	GenePrint_10_v1.1		

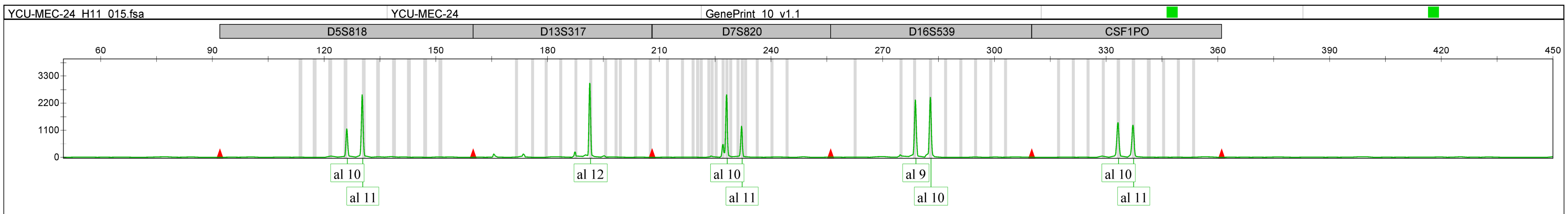

YCU-MEC-24_H11_015.fsa	YCU-MEC-24	GenePrint_10_v1.1		

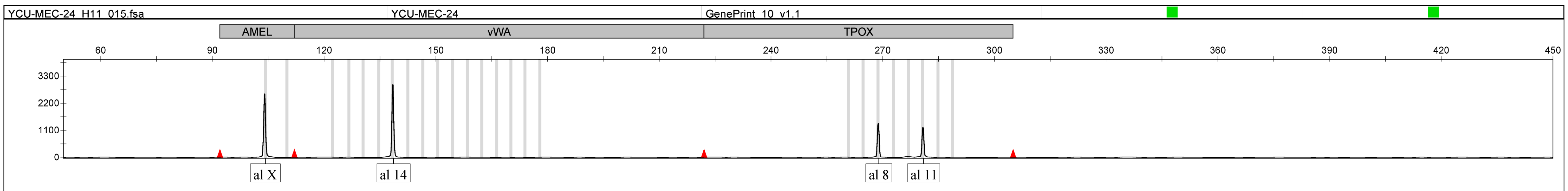

Sample File	Sample Name	Panel	OS	SQ
YCU-MEC-24X_A12_002.fsa	YCU-MEC-24X	GenePrint_10_v1.1		

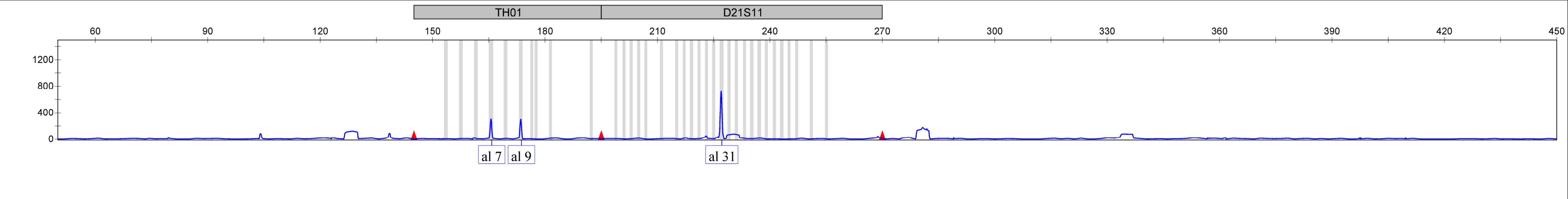

Sample File	Sample Name	Panel	OS	SQ
YCU-MEC-24X_A12_002.fsa	YCU-MEC-24X	GenePrint_10_v1.1		

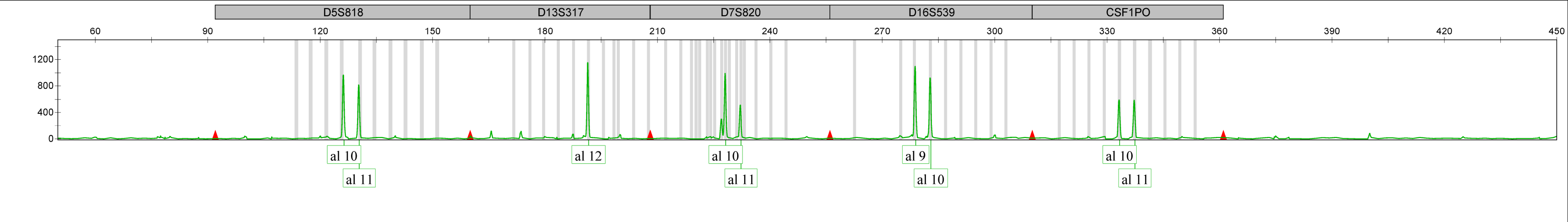

Sample File	Sample Name	Panel	OS	SQ
YCU-MEC-24X_A12_002.fsa	YCU-MEC-24X	GenePrint_10_v1.1		

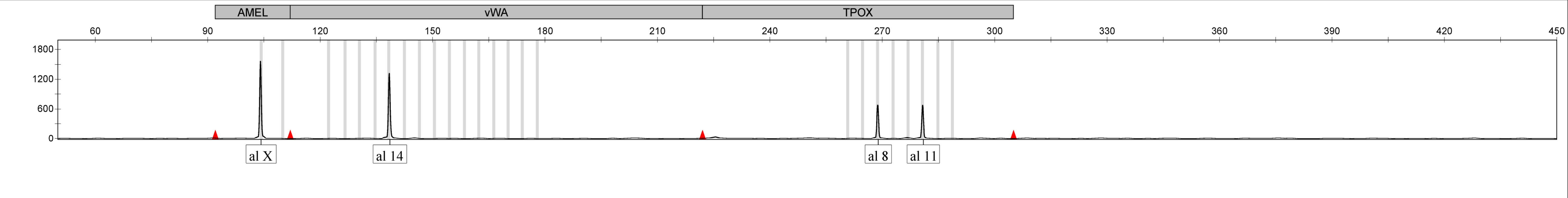

Sample File	Sample Name	Panel	OS	SQ
YCU-MYEC-16_primary_tumor_C11_005.fsa	YCU-MYEC-16_primary_tumor	GenePrint_10_v1.1		

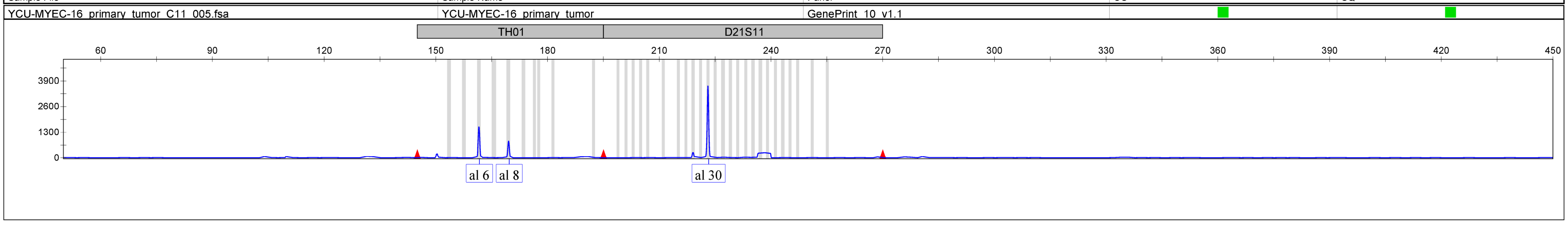

YCU-MYEC-16_primary_tumor_C11_005.fsa	YCU-MYEC-16_primary_tumor	GenePrint_10_v1.1

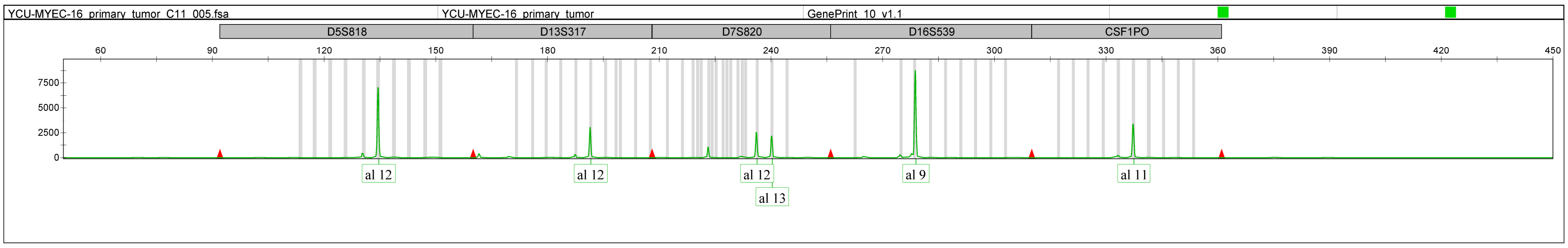

YCU-MYEC-16_primary_tumor_C11_005.fsa	YCU-MYEC-16_primary_tumor	GenePrint_10_v1.1

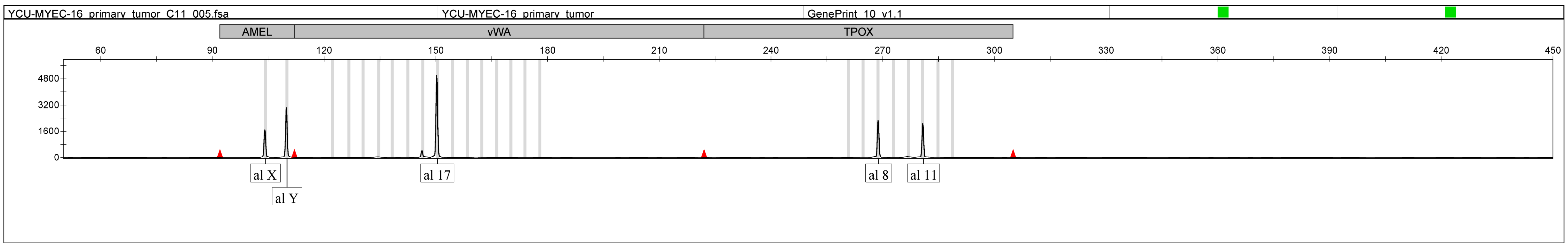

Sample File	Sample Name	Panel	OS	SQ
YCU-SDC-14_primary_tumor_A11_001.fsa	YCU-SDC-14_primary_tumor	GenePrint_10_v1.1		

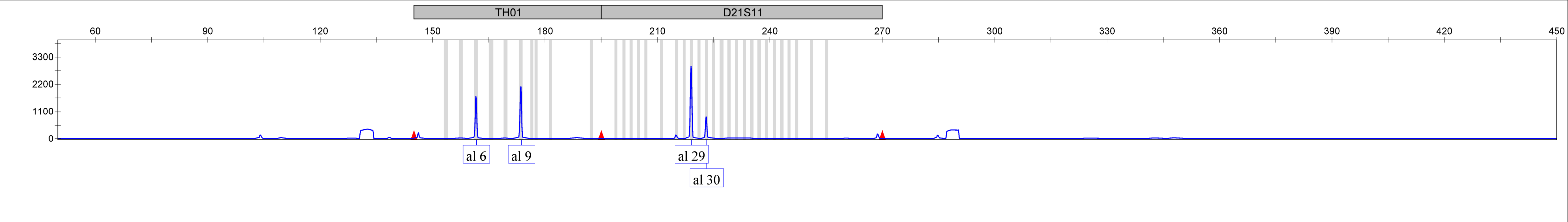

YCU-SDC-14_primary_tumor_A11_001.fsa	YCU-SDC-14_primary_tumor	GenePrint_10_v1.1		

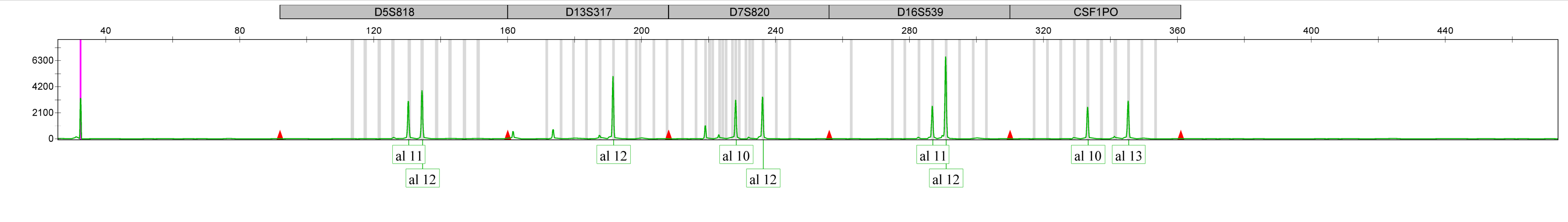

YCU-SDC-14_primary_tumor_A11_001.fsa	YCU-SDC-14_primary_tumor	GenePrint_10_v1.1		

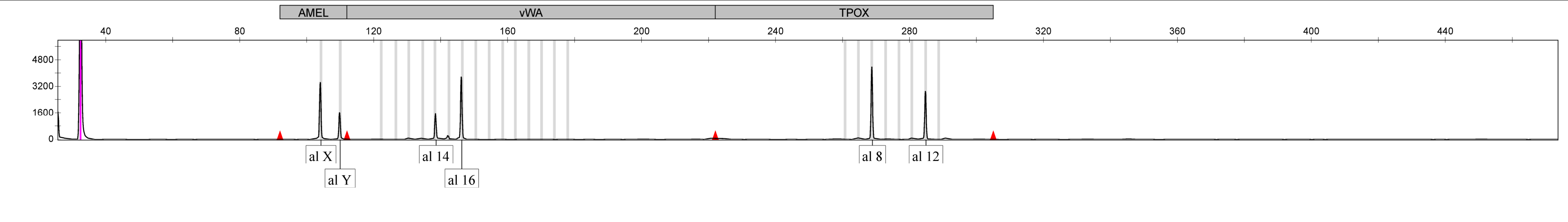

Sample File	Sample Name	Panel	OS	SQ
YCU-SDC-14 A02 002.fsa	YCU-SDC-14	GenePrint 10 v1.1		

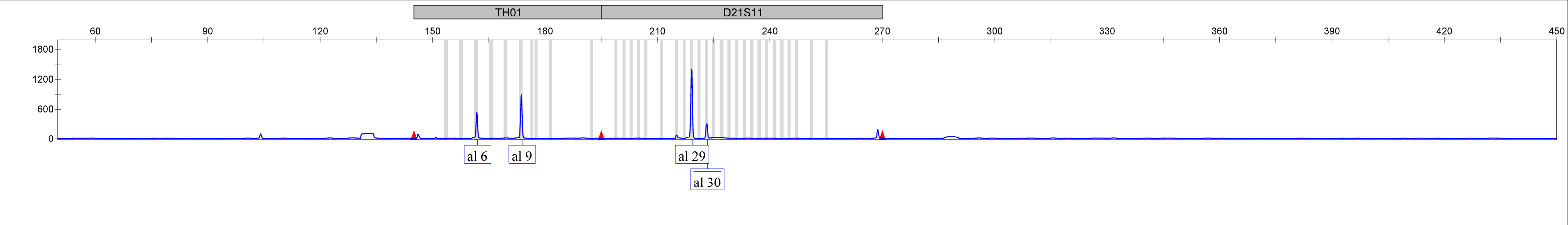

YCU-SDC-14 A02 002.fsa	YCU-SDC-14	GenePrint 10 v1.1		

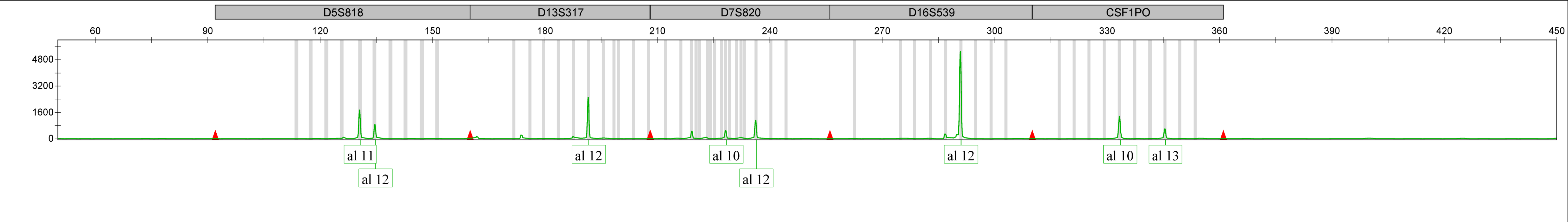

YCU-SDC-14 A02 002.fsa	YCU-SDC-14	GenePrint 10 v1.1		

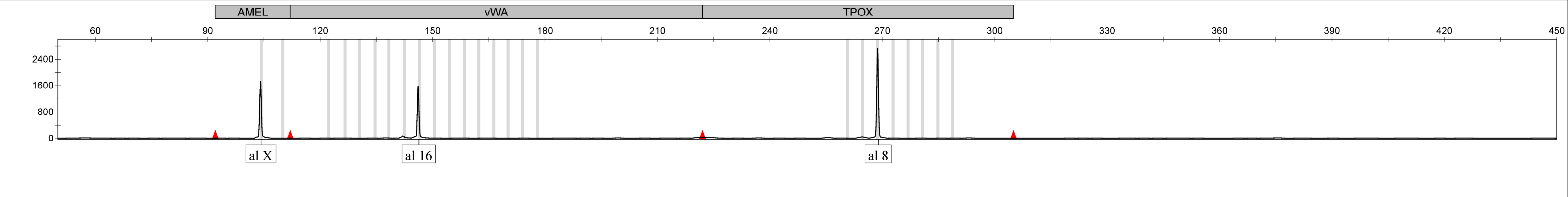

Applied Biosystems
GeneMapper Software 5

200925

Sample File	Sample Name	Panel	OS	SQ
YCU-SDC-14X_B01_003.fsa	YCU-SDC-14X	GenePrint_10_v1.1		

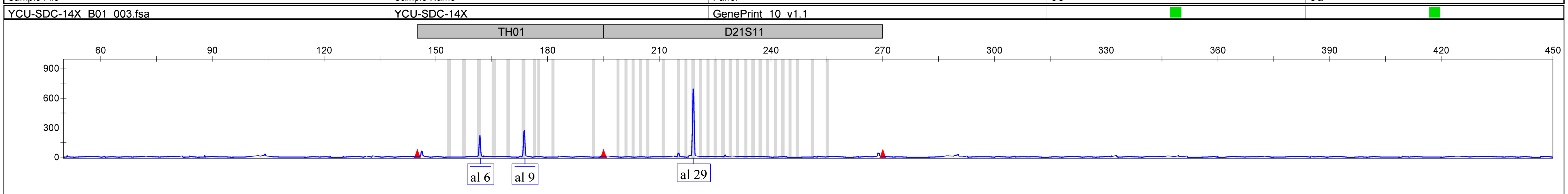

YCU-SDC-14X_B01_003.fsa	YCU-SDC-14X	GenePrint_10_v1.1		

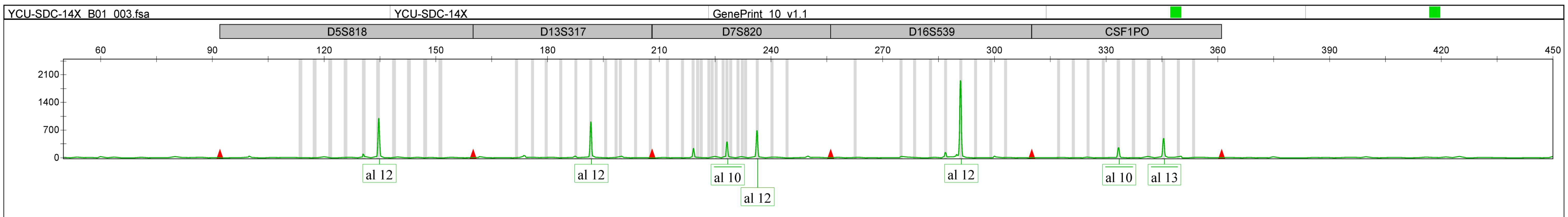

YCU-SDC-14X_B01_003.fsa	YCU-SDC-14X	GenePrint_10_v1.1		

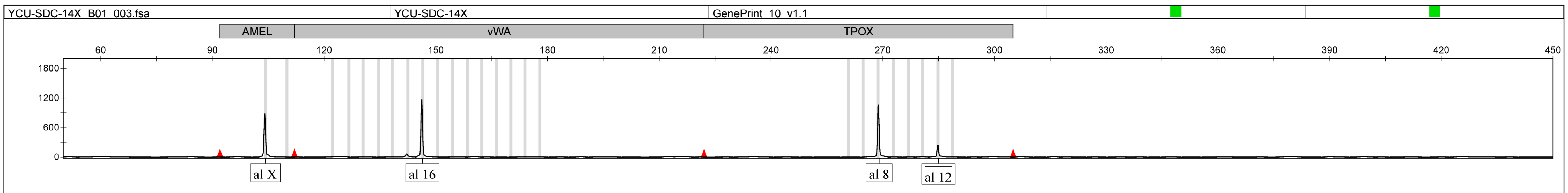

GeneMapper Software 5

201008

Sample File	Sample Name	Panel	OS	SQ
YCU-SDC-20_primary_tumor_G12_014.fsa	YCU-SDC-20_primary_tumor	GenePrint_10_v1.1		

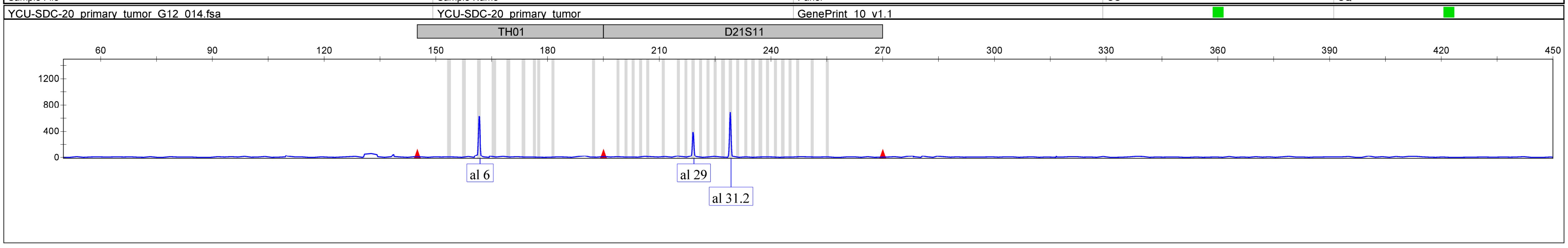

YCU-SDC-20_primary_tumor_G12_014.fsa	YCU-SDC-20_primary_tumor	GenePrint_10_v1.1		

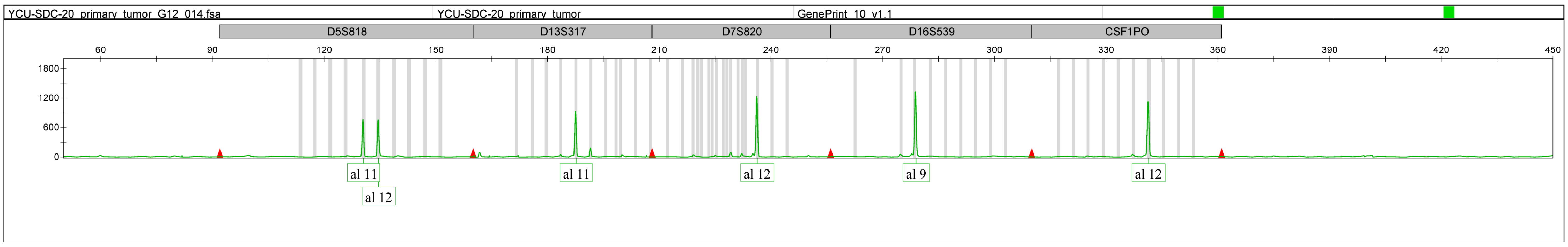

YCU-SDC-20_primary_tumor_G12_014.fsa	YCU-SDC-20_primary_tumor	GenePrint_10_v1.1		

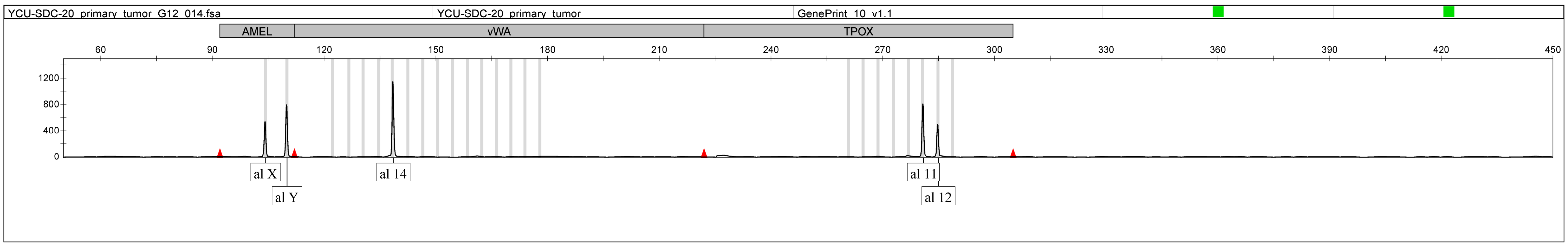

Applied Biosystems
GeneMapper Software 5

201008

Sample File	Sample Name	Panel	OS	SQ
YCU-SDC-20_F11_011.fsa	YCU-SDC-20	GenePrint_10_v1.1		

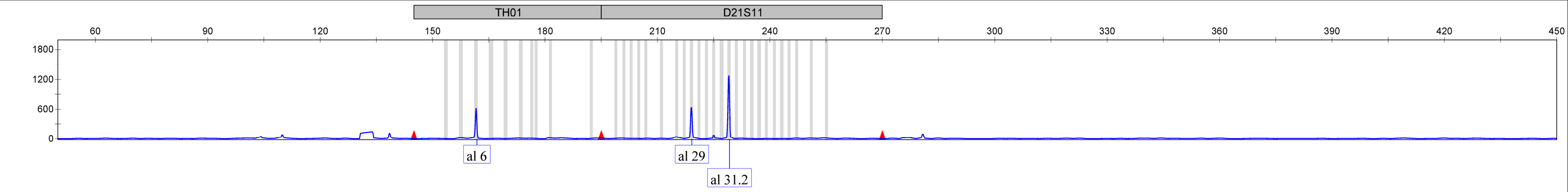

YCU-SDC-20_F11_011.fsa	YCU-SDC-20	GenePrint_10_v1.1		

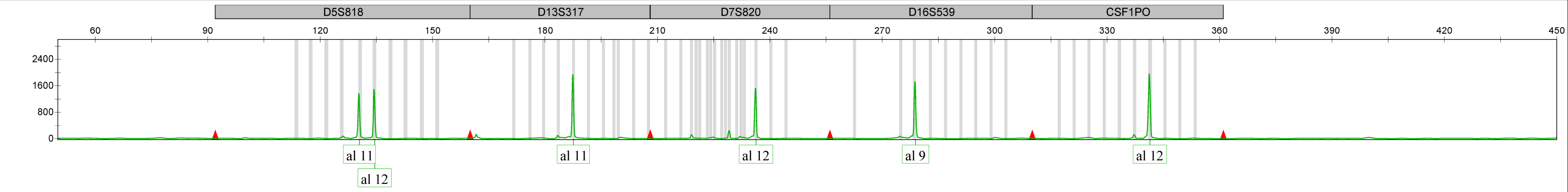

YCU-SDC-20_F11_011.fsa	YCU-SDC-20	GenePrint_10_v1.1		

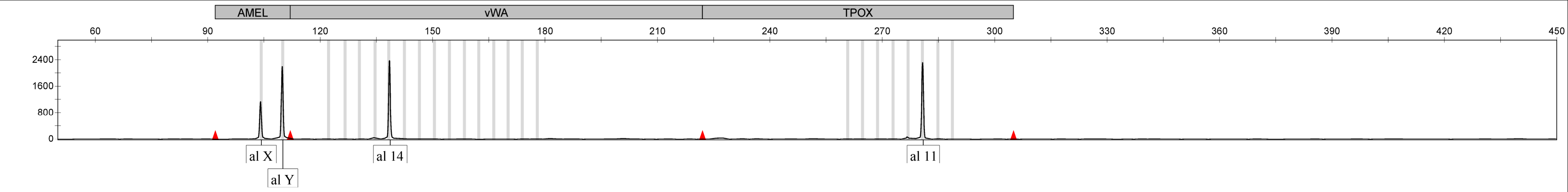

Sample File	Sample Name	Panel	OS	SQ
YCU-SDC-20X_G11_013.fsa	YCU-SDC-20X	GenePrint_10_v1.1		

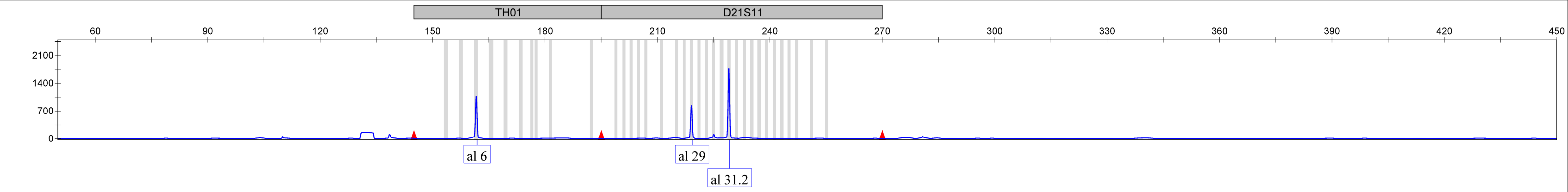

YCU-SDC-20X_G11_013.fsa	YCU-SDC-20X	GenePrint_10_v1.1		

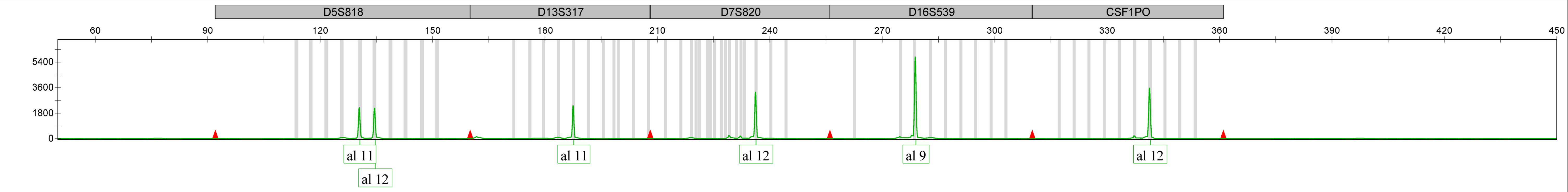

YCU-SDC-20X_G11_013.fsa	YCU-SDC-20X	GenePrint_10_v1.1		

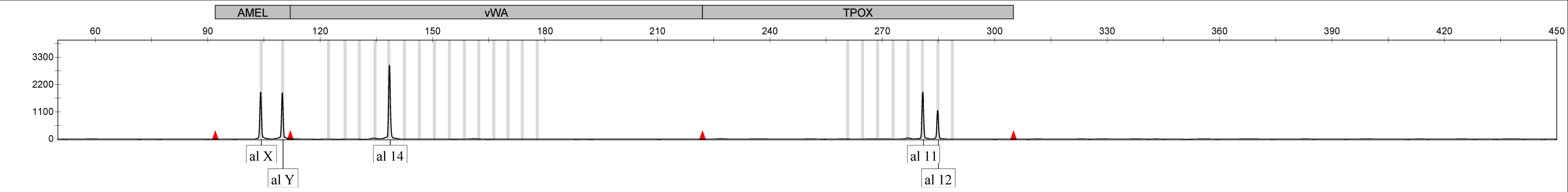

Sample File	Sample Name	Panel	OS	SQ
YCU-SDC-32_primary_tumor_E11_009.fsa	YCU-SDC-32_primary_tumor	GenePrint_10_v1.1		

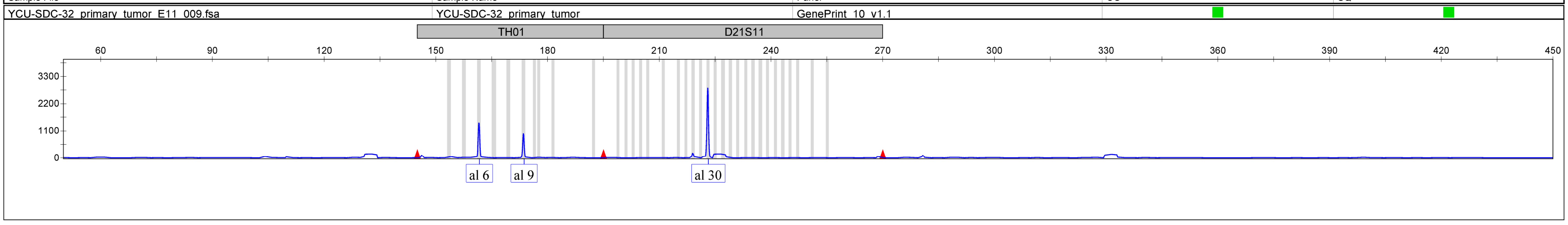

YCU-SDC-32_primary_tumor_E11_009.fsa	YCU-SDC-32_primary_tumor	GenePrint_10_v1.1		

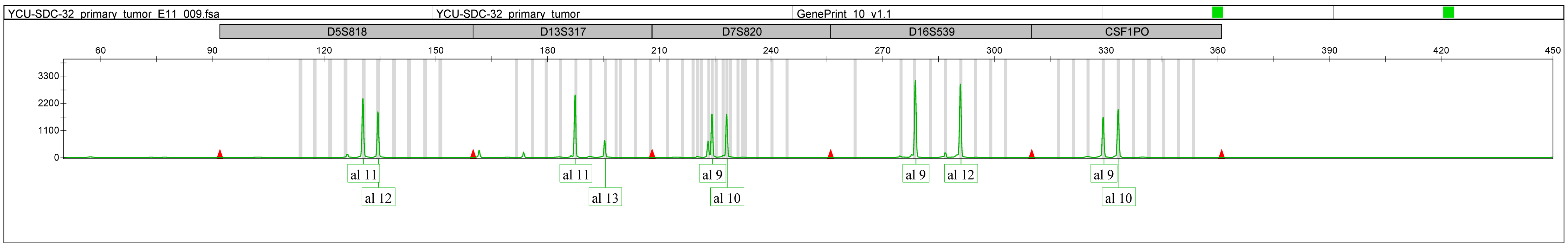

YCU-SDC-32_primary_tumor_E11_009.fsa	YCU-SDC-32_primary_tumor	GenePrint_10_v1.1		

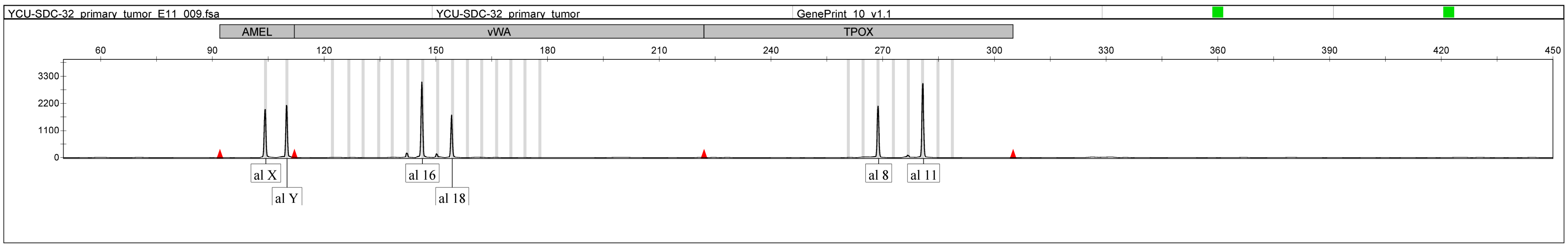

Sample File	Sample Name	Panel	OS	SQ
YCU-SDC-32_B12_004.fsa	YCU-SDC-32	GenePrint_10_v1.1		

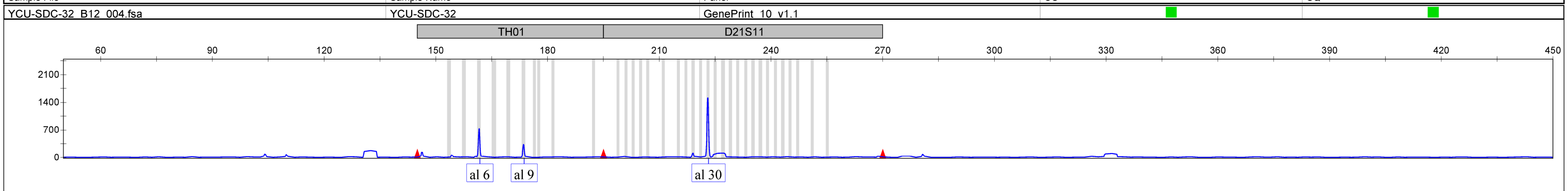

YCU-SDC-32_B12_004.fsa	YCU-SDC-32	GenePrint_10_v1.1		

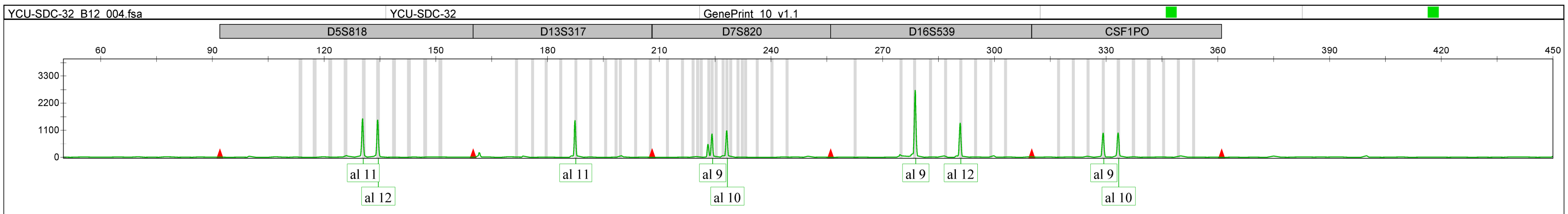

YCU-SDC-32_B12_004.fsa	YCU-SDC-32	GenePrint_10_v1.1		

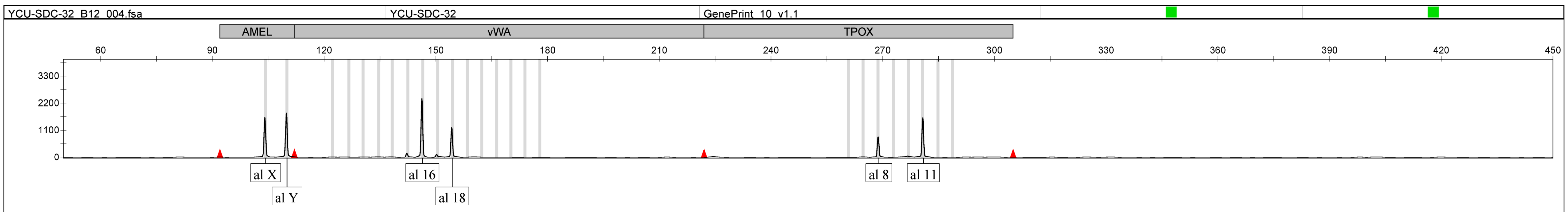

allele data

Locus	YCU-SDC-14 primary tumor		YCU-SDC-14		YCU-SDC-14X	
TH01	6	9	6	9	6	9
D21S11	29	30	29	30	29	
D5S818	11	12	11	12	12	
D13S317	12		12		12	
D7S820	10	12	10	12	10	12
D16S539	11	12	12		12	
CSF1PO	10	13	10	13	10	13
AMEL	X	Y	X		X	
vWA	14	16	16		16	
TPOX	8	12	8		8	12

Locus	YCU-SDC-20 primary tumor		YCU-SDC-20		YCU-SDC-20X	
TH01	6		6		6	
D21S11	29	31.2	29	31.2	29	31.2
D5S818	11	12	11	12	11	12
D13S317	11		11		11	
D7S820	12		12		12	
D16S539	9		9		9	
CSF1PO	12		12		12	
AMEL	X	Y	X	Y	X	Y
vWA	14		14		14	
TPOX	11	12	11		11	12

Locus	YCU-MEC-24 primary tumor		YCU-MEC-24		YCU-MEC-24X	
TH01	7	9	7	9	7	9
D21S11	31		31		31	
D5S818	10	11	10	11	10	11
D13S317	12		12		12	
D7S820	10	11	10	11	10	11
D16S539	9	10	9	10	9	10
CSF1PO	10	11	10	11	10	11
AMEL	X		X		X	
vWA	14		14		14	
TPOX	8	11	8	11	8	11

Locus	YCU-SDC-32 primary tumor		YCU-SDC-32	
TH01	6	9	6	9
D21S11	30		30	
D5S818	11	12	11	12
D13S317	11	13	11	
D7S820	9	10	9	10
D16S539	9	12	9	12
CSF1PO	9	10	9	10
AMEL	X	Y	X	Y
vWA	16	18	16	18
TPOX	8	11	8	11
